# RBM4 dictates ESCC cell fate switch from cellular senescence to glutamine-addiction survival through inhibiting LKB1-AMPK-axis

**DOI:** 10.1038/s41392-023-01367-x

**Published:** 2023-04-21

**Authors:** Lei Chen, Wenjing Zhang, Dan Chen, Quan Yang, Siwen Sun, Zhenwei Dai, Zhengzheng Li, Xuemei Liang, Chaoqun Chen, Yuexia Jiao, Lili Zhi, Lianmei Zhao, Jinrui Zhang, Xuefeng Liu, Jinyao Zhao, Man Li, Yang Wang, Yangfan Qi

**Affiliations:** 1 Institute of Cancer Stem Cells and the Second Affiliated Hospital of Dalian Medical University, Dalian Medical University, Dalian, 116044 China; 2 Department of Pathology, the First Affiliated Hospital of Dalian Medical University, Dalian, 116011 China; 3 Department of Oncology, the Second Affiliated Hospital of Dalian Medical University, Dalian, 116023 China; 4 Department of Thoracic Surgery, the First Affiliated Hospital of Dalian Medical University, Dalian, 116011 China; 5 Research Center, the Fourth Hospital of Hebei Medical University, Shijiazhuang, 050011 China

**Keywords:** Cancer, Cell biology

## Abstract

Cellular senescence provides a protective barrier against tumorigenesis in precancerous or normal tissues upon distinct stressors. However, the detailed mechanisms by which tumor cells evade premature senescence to malignant progression remain largely elusive. Here we reported that RBM4 adversely impacted cellular senescence to favor glutamine-dependent survival of esophageal squamous cell carcinoma (ESCC) cells by dictating the activity of LKB1, a critical governor of cancer metabolism. The level of RBM4 was specifically elevated in ESCC compared to normal tissues, and RBM4 overexpression promoted the malignant phenotype. RBM4 contributed to overcome H-RAS- or doxorubicin-induced senescence, while its depletion caused P27-dependent senescence and proliferation arrest by activating LKB1-AMPK-mTOR cascade. Mechanistically, RBM4 competitively bound LKB1 to disrupt the LKB1/STRAD/MO25 heterotrimeric complex, subsequently recruiting the E3 ligase TRIM26 to LKB1, promoting LKB1 ubiquitination and degradation in nucleus. Therefore, such molecular process leads to bypassing senescence and sustaining cell proliferation through the activation of glutamine metabolism. Clinically, the ESCC patients with high RBM4 and low LKB1 have significantly worse overall survival than those with low RBM4 and high LKB1. The RBM4 high/LKB1 low expression confers increased sensitivity of ESCC cells to glutaminase inhibitor CB-839, providing a novel insight into mechanisms underlying the glutamine-dependency to improve the efficacy of glutamine inhibitors in ESCC therapeutics.

## Introduction

Esophageal carcinoma is one of the most aggressive cancers, which is the seventh in incidence and sixth leading cause of cancer death worldwide.^1^ More than 70% of global esophageal cancer cases occur in China.^2^ Esophageal squamous cell carcinoma (ESCC) accounts for ~90% of all diagnosed cases in China. Currently, limited clinical approaches have been identified for the early diagnosis and treatment of ESCC, leading to an extremely poor prognosis, with the 5-year survival rate ranging from only 10% to 25% .^3^ Genome-wide screening has identified multiple genetic alterations in ESCC, including the amplification of cyclin D1, which could drive Glutamine-addiction.^4,5^ Glutamine-addiction or Glutamine (Gln) metabolism has been shown to play critical roles in sustaining tumor rapid growth and metastasis.^6–8^ Briefly, Glutamine-addiction provides energetic and synthetic needs for cancer cells, maintaining the redox homeostasis and biomass accumulation as a central metabolic hub.^8,9^ Recently, increased Glutamine metabolism was reported in two independent studies when comparing ESCC with the normal esophageal tissues,^5,10^ implying that targeting Glutamine-addiction might offer a promising avenue to treat human ESCC. Therefore, exploring the molecular mechanisms that trigger Glutamine-dependency may improve the efficacy of therapeutic target to glutamine metabolism.

Serine/threonine kinase STK11 (LKB1) was commonly known as a crucial tumor suppressor with a high frequency of genetic alterations in multiple subtypes of non-small cell lung carcinomas (NSCLC), cervical carcinomas and breast cancer.^11–15^ Accumulating evidence demonstrated that LKB1-loss leads to the progression of distinct cancers, such as NSCLC,^12^ cervical cancer,^14^ pancreatic cancer,^16^ melanoma,^17^ the myeloproliferative neoplasms^18^ and ESCC.^19,20^ The tumor-suppressive activity of LKB1 is mainly controlled by its phosphorylated substrate AMPK, which is a master regulator in cell survival and metabolic processes, including Glutamine-addiction.^21,22^ AMPK was reported to phosphorylate PPARδ, inhibiting the transcription of glutamine transporter SLC1A5.^23^ Moreover, LKB1 deletion could render KRAS-mutant NSCLC or polycystic kidney disease, relying on glutamine metabolism in a context-dependent manner.^22,24,25^ Meanwhile, the activation of LKB1-AMPK pathway was tightly associated with autophagy, apoptosis and cell senescence in response to genetic or environmental stimulus.^26,27^

During tumor initiation and progression, cellular senescence occurred in precancerous tissues with molecular lesions resulted from telomere shortening, oncogene stress and oxidative stress, thereby inducing persistent cell cycle arrest, promoting immune clearance and ultimately avoiding malignant expansion of cancer cells.^28,29^ Senescent cells usually present numerous common phenotypes, such as flattened and enlarged changes in cell morphology, elevated senescence-associated β-galactosidase (SA-β-gal) activity, changes in transcriptional levels of multiple cell-cycle inhibitors, increased levels of ROS, as well as metabolic reprogramming.^30–32^ It’s generally accepted that cancer cells could bypass or evade senescence to re-entry cell cycle and spread malignantly.^33,34^ Importantly, cell cycle progression appeared to be linked with active anabolic metabolism, depending on large amounts of glucose and glutamine utilization to meet the bioenergetic and biosynthetic demand.^35,36^ Although glutamine was required by cancer cells to evade therapy-induced senescence,^37^ the metabolic dependencies and the underlying molecular mechanisms of the cellular escape from senescence are still less well understood.

RNA-binding proteins (RBPs) are a family of proteins that play vital roles in the regulation of gene expression through the canonical function in manipulating the creation or process of RNAs, or the non-canonical function independent of RNA binding domains. The malfunction of RBPs results in various diseases, including human cancer.^38,39^ RNA-binding motif 4 (RBM4) has been identified as a potential tumor suppressor to inhibit cancer progression in multiple cancers.^39–42^ In addition to its classic function as a splicing factor, RBM4 also participates in regulating translation and RNA stability.^43–45^ However, the potential role of RBM4 in ESCC remains largely unknown. Here we report a surprising finding that RBM4 functions as an oncogene in ESCC by protecting ESCC cells from senescence to sustain Glutamine-dependent survival. Mechanistically, RBM4 competitively bound to LKB1 to disrupt the formation of LKB1/STRAD/MO25 heterotrimeric complex. Meanwhile, RBM4 recruited the E3 ligase TRIM26 to LKB1, promoting the ubiquitination of LKB1 and its subsequent degradation, thereby conferring the glutamine addiction to RBM4-high ESCC. Conversely, RBM4 depletion stabilized LKB1 to increase p-AMPK, thus inhibiting mTOR activity and inducing P27-dependent senescence. Our study therefore uncovered a novel tumor-promoting function of RBM4 in ESCC, offering a new potential biomarker for glutamine-dependent therapeutics.

## Results

### RBM4 exhibits oncogenic activity in ESCC

**Fig. 1:**
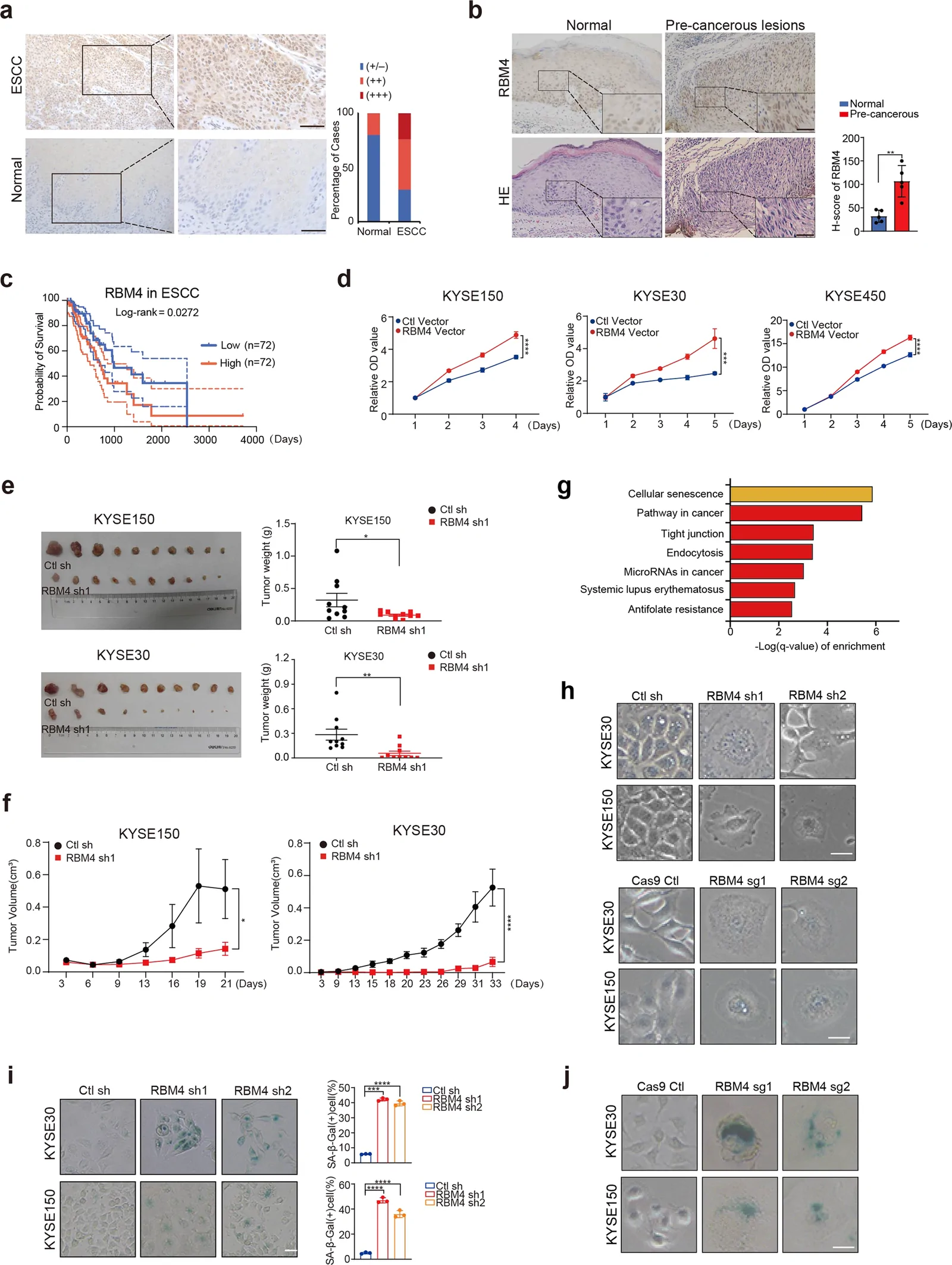
RBM4 exhibits oncogenic activity in ESCC. a RBM4 protein levels were measured in 75 pairs of ESCC and normal esophageal tissues by immunohistochemistry staining of tissue microarray. Quantification of the percentage of cases exhibited negative, 1 + , 2+ or 3+ IHC staining of RBM4 was plotted. “−” indicates negative staining; “+” means weak positive staining; “++” denotes moderately strong positive staining, and “+++” indicates strong positive staining. Scale bar = 50 μm. **b** Immunohistochemistry and HE staining of normal esophageal tissues and pre-cancerous lesions of ESCC were shown. Scale bar = 10 μm. Quantitative analysis of RBM4 expression was done by Image J program. Plotted are the mean ± SD from five pairs of human samples, with *P < 0.05 as determined by paired Student *t*-test. *H*-score = ∑(pi × i) = (percentage of weak intensity × 1)+(percentage of moderate intensity × 2) + (percentage of strong intensity × 3), pi indicates the percentage of positive signal pixel area/number of positive tumor cells, i represents the coloring intensity. **c** Kaplan–Meier curve with corresponding 95% confidence intervals (CI) showing overall survival of ESCC patients with high or low RBM4 expression based on immunohistochemical microarray analysis. Dotted red/blue indicates the upper and lower confidence limits. Significance was assessed with Mantel-Cox log-rank (*P* = 0.0272) test. The hazard ratio for risk of death in RBM4-low versus RBM4- high ESCC patients is 0.5636 (95%CI = 0.375-0.9412). **d** Cell viabilities of RBM4-overexpressed KYSE150, KYSE30 and KYSE450 cells were measured by the CCK8 growth curve assays. Three experiments were carried out with mean ± SD of relative cell viability plotted. *P*-values were determined by two-way repeated measures ANOVA, ***P < 0.001, ****P < 0.0001. **e**, **f** Nude mice were subcutaneously inoculated with KYSE150 or KYSE30 cells with stable knockdown of RBM4 or control respectively. Images of the subcutaneous xenograft tumors were shown in **e**. Tumor weight (**e**) and tumor volumes (**f**) of each group was measured and quantified at the indicated time. *P-*values were determined by Student’s *t*-test (**e**) or two-way repeated measures ANOVA (**f**). (*n* = 10, error bars indicate mean ± SEM, *P < 0.05, **P < 0.01.) **g** KEGG pathway analysis of RNA-seq with three independent biological replicates using KYSE150 cells with stable depletion of RBM4 compared to control vector. Statistical analysis was performed with the false discovery rate (FDR) method after Benjamini-Hochberg correction for multiple-testing. KEGG terms for biological process with FDR < 0.05 were accepted as a significant enrichment. **h** The morphology changes of KYSE30 and KYSE150 cells with stable knockdown of RBM4 by shRNA (upper panel) or knockout of RBM4 established by CRISPR-Cas9 system (lower panel). Scale bar = 25 μm. **i** β-gal staining of KYSE30 and KYSE150 cells with stable depletion of RBM4 by shRNA. Three experiments were carried out with mean ± SD of β-gal positive cells plotted. ****P < 0.0001 (*P-*values were determined by One-way ANOVA with Dunnett multiple comparisons). Scale bar = 25 μm. **j** β-gal staining of RBM4-knockout KYSE30 and KYSE150 cells generated through CRISPR-Cas9 system. Scale bar = 25 μm

RNA-binding proteins (RBPs) play important roles in multiple cancers, however, the functions of RBPs in ESCC have not been fully characterized. To systematically identify the role of RBPs in ESCC, we analyzed the expression levels of 124 known RBPs in TCGA ESCC dataset. Surprisingly, RBM4, a known tumor-suppressive RBP in multiple cancers,^39,41,42,46,47^ was one of the top hits that was dramatically elevated in ESCC samples as compared to normal tissues (Supplementary Fig. 1a). We also analyzed the microarray data deposited in NCBI’s GEO database and found RBM4 is highly expressed in ESCC in comparison with the normal esophageal squamous epithelium according to the GSE161533 and GSE26886 datasets (Supplementary Fig. 1a). To confirm this observation, we directly examined the expression level of RBM4 by immunohistochemistry staining of 75 paired ESCC and normal clinical samples (Fig. 1a). The extra-strong staining of RBM4 was detected in 18 out of 75 (24%) ESCC samples, and strong staining was examined in 35 tumors (47%). In contrast, most normal esophageal tissues (60 of 75, 80%) exhibited weak staining or undetectable of RBM4. Such elevated expression of RBM4 was also observed in pre-cancerous lesions of ESCC (Fig. 1b). Kaplan-Meier analysis revealed a significant correlation between low RBM4 levels and improved overall survival in patients with ESCC through IHC score in the tissue array (Fig. 1c). Together, RBM4 is notably increased in ESCC in independent patient cohorts, suggesting that RBM4 might function as a tumor-promoting RBP in ESCC.

To directly test the possible involvement of RBM4 in ESCC, we first measured the RNA levels of RBM4 in a panel of ESCC cell lines and found that RBM4 was significantly increased in ESCC cells as compared to normal esophageal epithelial cells (NE2 and NE3) (Supplementary Fig. 1b). We then stably overexpressed or knocked down RBM4 in distinct ESCC cells by infecting them with lentiviral particles (Supplementary Fig. 1c, d). RBM4 overexpression significantly promoted the growth and proliferation of KYSE150, KYSE30 and KYSE450 cells (Fig. 1d and Supplementary Fig. 1e). In addition, RBM4 accelerated the migration of ESCC cells as judged by wound-healing and transwell assays (Supplementary Fig. 1f, g). Conversely, depletion of RBM4 significantly suppressed the proliferation of ESCC cells (Supplementary Fig. 1h, i). Moreover, knockdown of RBM4 inhibited cell migration of multiple ESCC cells (Supplementary Fig. 1j). Consistent with the in vitro cell culture data, the RBM4-depleted xenograft tumors grew extremely slow compared to control tumors (Fig. 1e, f), indicating that reduction of RBM4 significantly suppresses ESCC progression in vivo.

To further investigate the impact of RBM4 on ESCC progression, we analyzed the transcriptional alterations of RBM4-depleted KYSE150 cells by the mRNA-seq assay. KEGG analysis of differentially expressed genes showed that the RBM4-regulated genes were enriched in senescence-related pathway (Fig. 1g). Consistent with this finding, both RBM4-depleted KYSE150 and KYSE30 cells showed enlarged and flattened morphologic changes (Fig. 1h), and had dramatically increased SA-β-gal staining intensity (Fig. 1i), which is a distinctive trait of cellular senescence. We also generated RBM4 knockout ESCC cells using CRISPR-Cas9 system, and applied two siRNAs to knockdown RBM4 in ESCC cells (Supplementary 2a, b), which were subsequently subjected to morphological observation and SA-β-gal staining assays. The results showed that RBM4 knockout by Cas9/sgRNA or knockdown by siRNA also led to the flattened and enlarged morphology of ESCC cells and elevated the percentage of SA-β-gal positive cells (Fig. 1h, j and Supplementary 2c, d), which is in line with the results of RBM4 shRNAs, supporting the conclusion that RBM4 knockdown induced ESCC cellular senescence. Since cellular senescence suppresses tumorigenesis by stably arresting proliferation, we proposed that RBM4 may be involved in ESCC progression through regulating senescence.

### Loss of RBM4 induces ESCC cellular senescence and its overexpression contributes to bypass premature senescence

**Fig. 2:**
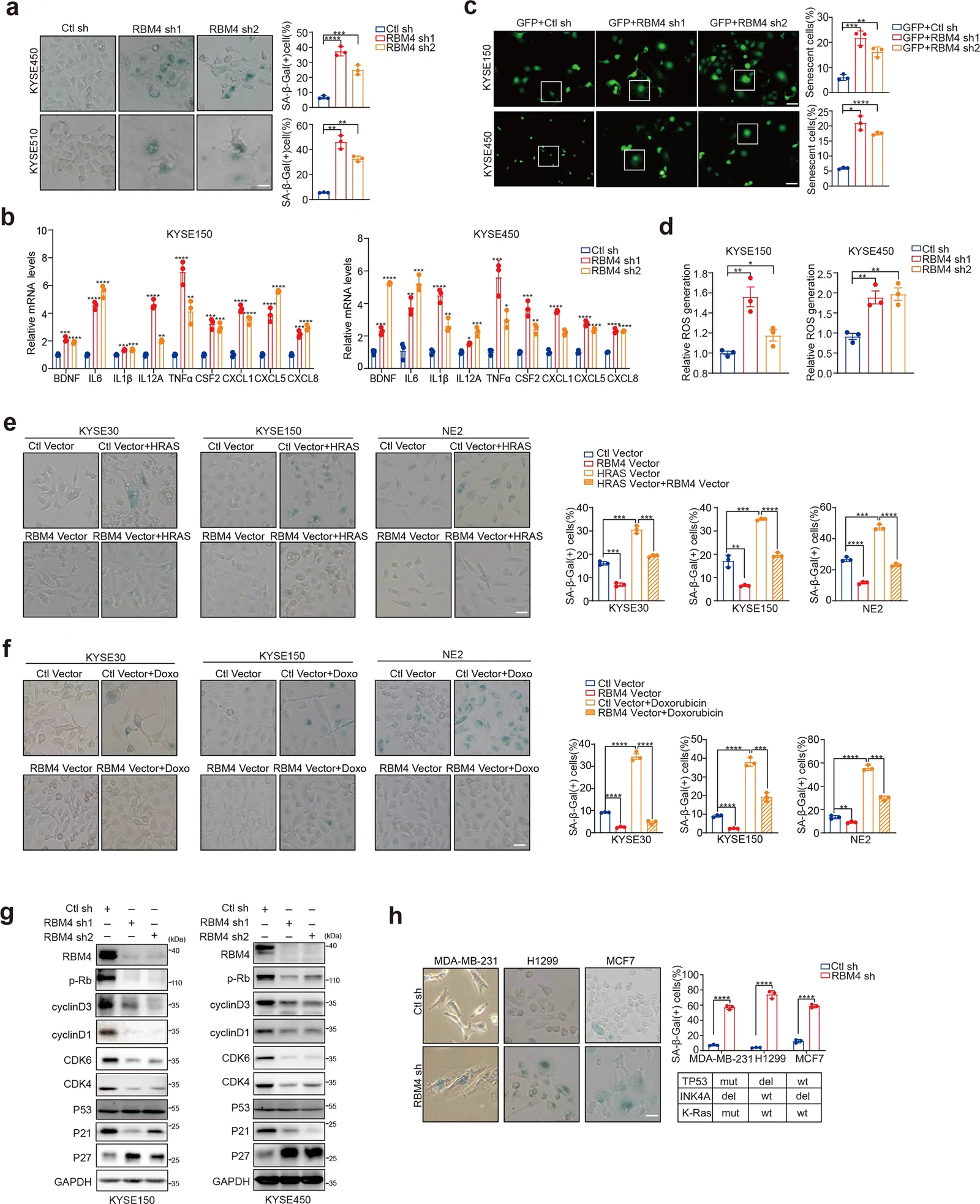
RBM4 depletion induces senescence-like phenotype and its overexpression contributes to bypass premature senescence. **a** β-gal staining of KYSE450 and KYSE510 cells with stable depletion of RBM4. Three experiments were carried out with mean ± SD of β-gal positive cells plotted. *P*-values were determined by One-way ANOVA with Dunnett multiple comparisons. Scale bar = 25 μm. **b** The expression of senescence-associated secretory phenotype (SASP) factors in KYS150 and KYSE450 cells with stable depletion of RBM4 was examined with the qRT-PCR approach. *P*-values were determined by One-way ANOVA with Dunnett multiple comparisons. *n* = 3 per group. **c** ESCC cells expressing GFP were co-cultured with RBM4-depleted ESCC cells, and morphology changes of ESCC-GFP cells were observed. Scale bar = 50 μm. Mean ± SD of percentage of relative senescence cells were plotted. *P*-values were determined by One-way ANOVA with Dunnett multiple comparisons. *n* = 3 per group. **d** The relative ROS production was examined in KYSE150 and KYSE450 cells with depletion of RBM4. Three experiments were carried out with mean ± SD of relative ROS production was plotted. (*P*-values were determined by One-way ANOVA with Dunnett multiple comparisons). **e**, **f** β-gal staining of KYSE30, KYSE150 and NE2 cells with stable lentiviral transfection of RBM4 or empty vector with or without H-RAS overexpression (**e**) or Doxorubicin treatment (**f**). Three experiments were carried out with mean ± SD of β-gal positive cells plotted (*P-*values were determined by One-way ANOVA with Dunnett multiple comparisons). Scale bar = 25 μm. **g** The protein levels of RBM4, p-Rb, cyclin D3, cyclin D1, CDK6, CDK4, P53, P21, and P27 in ESCC cells with RBM4 depletion were examined with a western blot assay. **h** β-gal staining of different cancer cells with distinct genetic background in the presence or absence of RBM4. Three experiments were performed with mean ± SD of β-gal positive cells plotted (*P*-values were determined by Student’s *t*-test) and the status of P53, INK4A, and K-Ras was indicated. Scale bar = 25 μm. *P < 0.05, **P < 0.01, ***P < 0.001, ****P < 0.0001

To further verify the impact of RBM4 depletion on cellular senescence, we applied two stably transfected cell lines KYSE450 and KYSE510 cells to stain for β-gal and found RBM4 depletion also increased the proportion of β-gal positive cells (Fig. 2a). Senescent cells have been shown to display senescence associated secretory phenotype (SASP) by secreting pro-inflammatory factors, cytokines, and chemokines, which in turn leads to the senescence of adjacent cells.^34^ In line with this notion, the mRNA levels of various SASP factors were significantly upregulated in RBM4-depleted ESCC cells compared to control cells (Fig. 2b). In addition, when co-cultured with RBM4-depleted cells, normal GFP positive cells displayed flattened and enlarged morphologic changes (Fig. 2c). Such phenomenon was consistent with a significant increase in ROS production in KYSE150 and KYSE450 cells (Fig. 2d). The above results indicated that RBM4 expression is tightly linking to the occurrence or process of cellular senescence. Senescence bypass has been proposed to play an influential role in progression of pre-neoplastic lesions to cancer due to certain oncogenic pathways.^48–50^ It has been known that cells are prone to entry senescence upon the aberrant activation of Ras pathway, which was a key oncogenic event in carcinogenesis.^51–53^ Moreover, doxorubicin, a chemotherapeutic drug in clinical therapy, also has been demonstrated to induce cellular senescence.^54,55^ However, the mechanistic basis for how cancer cell or pre-cancerous cells override senescence remain to be obscure. Since RBM4 was highly expressed in tumor and pre-cancerous lesions compared to normal tissues and exhibited oncogenic activity in ESCC as showed in Fig. 1a–f and Supplementary Fig. 1, we next define whether RBM4 might play a role in promoting senescence bypass during ESCC progression. Rescue experiments were performed by overexpressing RBM4 in immortalized esophageal epithelial cells (NE2) or KYSE30 and KYSE150 cells during HRAS- or doxorubicin-induced senescence. As demonstrated in Fig. 2e, f, H-Ras overexpression or doxorubicin treatment evidently increased the proportion of SA-β-gal-positive KYSE30 and KYSE150 cells (Fig. 2e, f, Supplementary Fig. 2e), suggesting the reliability of cellular senescence model. Notably, the proportion of β-gal positive cells were decreased in RBM4-overexpressing NE2 and ESCC cells (Fig. 2e, f), implying the ability of RBM4 to facilitate cellular senescence bypass in the context of H-RAS activation or DNA damage stress.

Meanwhile, we noticed that RBM4 ablation led to the increased level of the cell cycle inhibitor P27, and decreased levels of cyclin D1, cyclin D3, CDK4, CDK6, and p-RB, however, the levels of P21 and P53, the well-known genes associated with pre-mature aging, were not elevated in ESCC cells (Fig. 2g and Supplementary Fig. 2f). In the IHC staining of xenografts with RBM4 depletion or control, P27 was strongly expressed accompanied by low expression of Ki67 (Supplementary Fig. 2g). Conversely, ectopic expression of RBM4 downregulated P27 expression and increased the levels of cyclin D1, cyclin D3, CDK4, CDK6, and p-RB in distinct ESCC cell lines (Supplementary Fig. 2h). Since senescence is commonly induced by P53-P21 or Rb-P16 (INK4A) pathways, we therefore applied different cancer cell lines containing distinct mutations of these key regulators, to further investigate which pathway accounts for the RBM4-depletion induced senescence. Interestingly, knockdown of RBM4 still induced senescence in cancer cells bearing mutations or deletions of P53, P16 (INK4A), and K-Ras (Fig. 2h), suggesting that depleted RBM4 might activate senescence via P27 pathway.

### RBM4-reduction directly regulates LKB1-AMPK cascade to trigger P27-dependent ESCC senescence

**Fig. 3:**
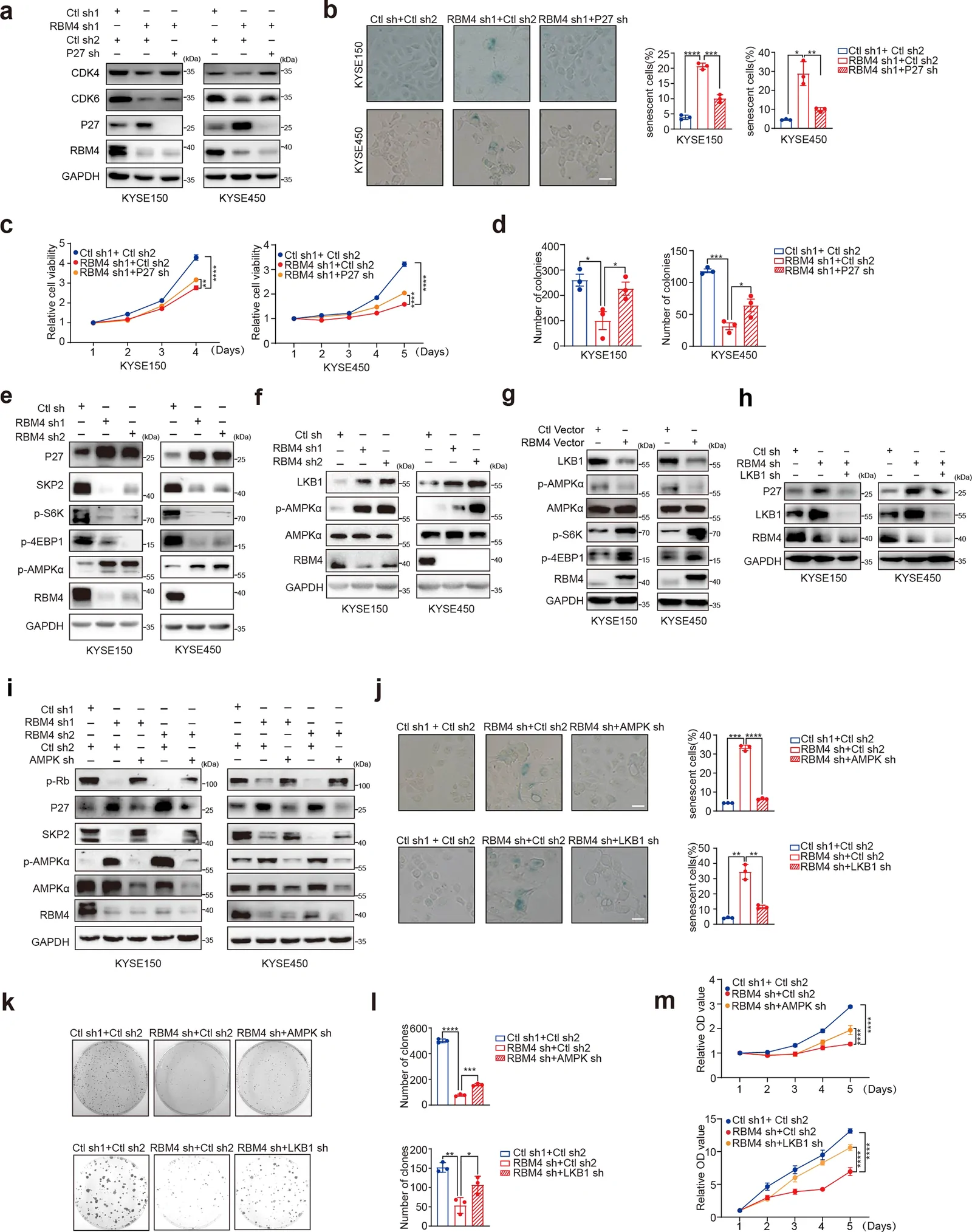
RBM4 reduction activates LKB1-AMPK-P27 pathway to induce cellular senescence and growth arrest. **a** Protein levels RBM4, CDK4, CDK6, and P27 were analyzed in RBM4-depleted ESCC cells with or without P27. **b** β-gal staining of KYSE150 and KYSE450 cells with stable knockdown of RBM4 in the presence or absence of P27. Three experiments were carried out with mean ± SD of β-gal positive cells plotted (*P*-values were determined by One-way ANOVA with Dunnett multiple comparisons). Scale bar = 25 μm. **c** The growth curve of RBM4-depleted KYSE150 or KYSE450 cells with or without depletion of P27 was measured by CCK8 assay. *P-*values were determined by two-way repeated measures ANOVA. *n* = 3 per group. **d** Colony formation assays of RBM4-depleted KYSE150 or KYSE450 cells with or without knockdown of P27 were performed. The mean ± SD of colony numbers was plotted (*P-*values were determined by One-way ANOVA with Dunnett multiple comparisons, *n* = 3 per group). **e** Protein levels of P27, SKP2, p-S6K, p-4EBP1, p-AMPK, and RBM4 were examined in RBM4-depleted KYSE150 and KYSE450 cells with a western blot assay. **f** Western blot was performed to measure the protein levels of LKB1, p-AMPK, AMPK, and RBM4 in KYSE150 and KYSE450 cells with or without RBM4. **g** Protein levels of LKB1, p-AMPK, AMPK, p-S6K, p-4EBP1, and RBM4 were examined in RBM4-overexpressed KYSE150 and KYSE450 cells using a western blot assay. **h** Western blot approach was performed to measure the protein levels of LKB1, RBM4, and P27 in RBM4-depleted ESCC cells with or without LKB1. **i** Protein levels of p-Rb, P27, SKP2, p-AMPK, AMPK, and RBM4 were analyzed in RBM4-depleted ESCC cells with or without AMPK. **j** β-gal staining of KYSE150 cells with stable knockdown of RBM4 in the presence or absence of AMPK or LKB1. Three experiments were carried out with mean ± SD of β-gal positive cells plotted (*P*-values were determined by One-way ANOVA with Dunnett multiple comparisons). Scale bar = 25 μm. **k**, **l** Colony formation assays of RBM4-depleted KYSE150 cells with or without knockdown of AMPK or LKB1 were performed. Three experiments were carried out with mean ± SD of colony numbers were plotted. *P*-values were determined by One-way ANOVA with Dunnett multiple comparisons. **m** Cell viability of RBM4-depleted KYSE150 cells with or without AMPK or LKB1 was measured by CCK8 assays. Three experiments were carried out with mean ± SD of relative cell viability plotted. *P-*values were determined by two-way repeated measures ANOVA. *P < 0.05, **P < 0.01, ***P < 0.001, ****P < 0.0001

In order to gain insights into the mechanism underlying the connection between RBM4 and ESCC cell fate decision, rescue experiments were performed by knocking down P27 in RBM4-depleted KYSE150 and KYSE450 cells. P27 reduction notably reversed the RBM4-depletion induced ESCC cellular senescence as judged by the downregulated CDK4 and CDK6 in a western blot assay (Fig. 3a), and decreased β-gal positive cell numbers relative to RBM4 knockdown alone (Fig. 3b). Our data imply that P27 accumulation is a principle causative factor in senescence triggered by RBM4 depletion. Moreover, P27 knockdown partially rescued RBM4-downregulation-induced growth inhibition in KYSE150 and KYSE450 cells as judged by growth curve and colony formation assays (Fig. 3c, d and Supplementary Fig. 2i), suggesting a non-exclusive mediator role of P27 in this process. Taken together, our data indicate that P27 elevation contributes to RBM4-deficency-induced ESCC cellular senescence and growth arrest.

We next sought to mechanistically investigate how RBM4 regulates P27 expression to facilitate ESCC cells evading senescence. We found depletion of RBM4 markedly decreased the ubiquitination of P27 as compared to the control (Supplementary Fig. 2j), suggesting that RBM4 might regulate the protein degradation of P27 through the ubiquitin pathway. As reported previously, SKP2 containing SCF (SKP1-cullin1-F-box protein) E3 ubiquitin ligase is able to degrade P27 through polyubiquitination.^56^ We thus measured the level of SKP2 in RBM4 stably depleted KYSE150 and KYSE450 ESCC cells, and revealed that the expression of SKP2 was evidently decreased by RBM4-depletion (Fig. 3e). Since the level of SKP2 could be modulated by AMPK through mTORC1 or Foxo3 pathways,^57^ we subsequently analyzed AMPK and mTOR signaling and found that depletion of RBM4 promoted the phosphorylation of AMPK, thereby inhibiting the phosphorylation of S6K and 4EBP1, which was activated by mTORC1 (Fig. 3e). However, the Foxo3 pathway was not evidently affected by knockdown of RBM4, as the localization of p-Foxo3 was not changed upon RBM4-depletion (Supplementary Fig. 2k). Therefore. our data suggest that RBM4 ablation-induced senescence might be dependent on accumulated P27 that resulted from AMPK-mTORC1-SKP2 pathway activation.

LKB1 is a major kinase that phosphorylates AMPK.^21^ Consistently, knockdown of RBM4 noticeably increased the level of LKB1 and p-AMPK, whereas overexpression of RBM4 markedly decreased the level of LKB1, p-AMPK, activating the downstream mTORC1 targets, S6K and 4EBP1 (Fig. 3f, g). In concert with this, RBM4 knockout by Cas9/sgRNA or knockdown by siRNA also upregulated the levels of LKB1, p-AMPK, P27 and inhibited the expression of positive regulators of cell cycle (Supplementary Fig. 3a, b). Furthermore, RBM4 depletion- induced P27 accumulation was notably reversed by LKB1 knockdown in ESCC cells (Fig. 3h). In line with this, the reduction of AMPK rescued the changes in the expression level of P27, SKP2 and cell cycle arrest marker p-Rb induced by RBM4 depletion (Fig. 3i). Importantly, the RBM4 depletion-induced senescence phenotype was suppressed by knocking down AMPK or LKB1 as judged by the β-gal staining assay (Fig. 3j). The RBM4 ablation-induced growth inhibition of ESCC cells was also in part overturned by the depletion of AMPK or LKB1 as shown in the colony formation and growth curve experiment (Fig. 3k–m), suggesting that RBM4-depletion activates LKB1-AMPK-P27 cascade, which triggers senescence, thereby inhibiting the proliferation of ESCC cells.

### RBM4 disrupts LKB1/STRAD/MO25 heterotrimeric complex by competitively binding to LKB1

**Fig. 4:**
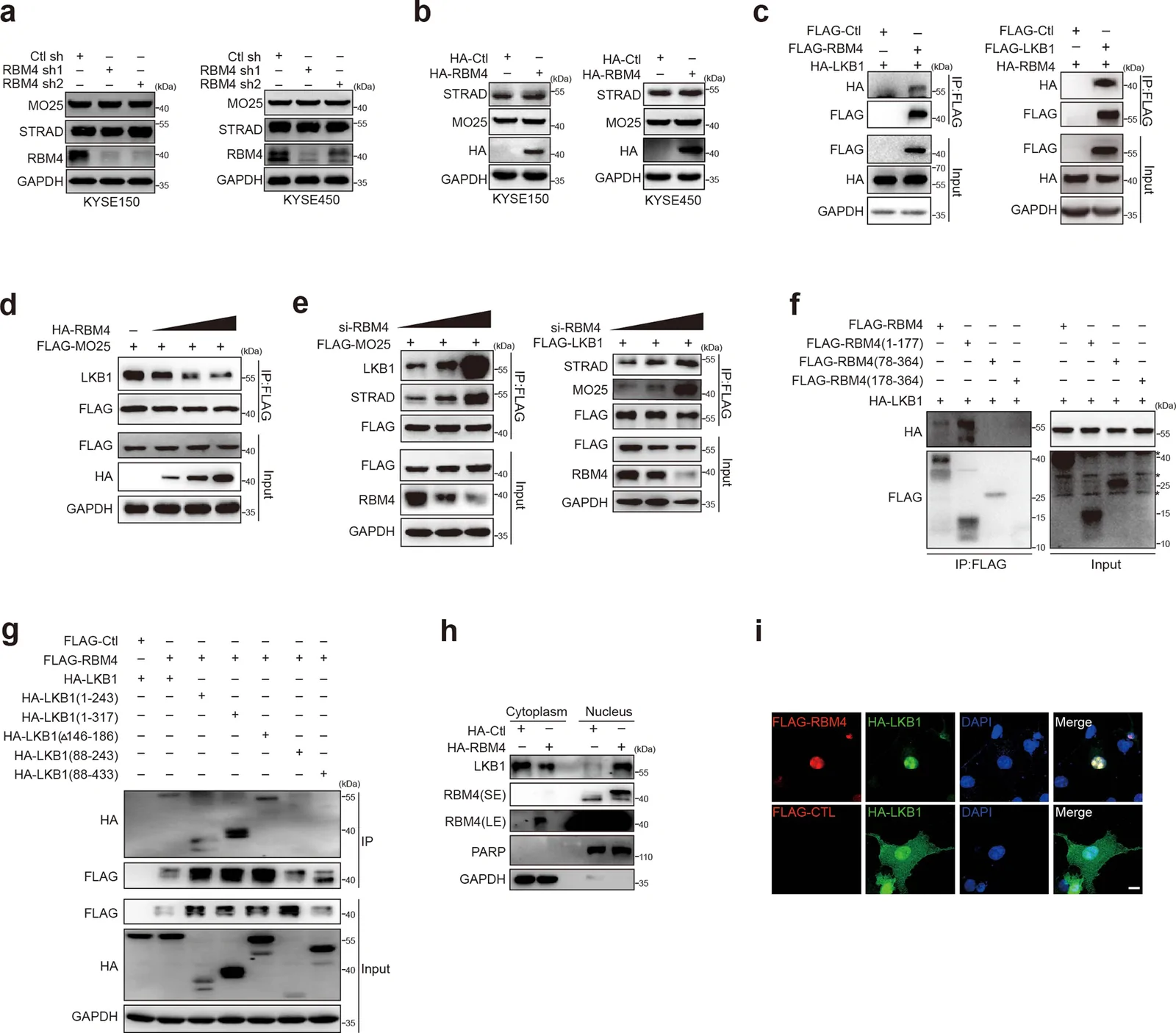
RBM4 disrupts LKB1/STRAD/MO25 heterotrimeric complex by competitively binding to LKB1. **a**, **b** Protein levels of MO25, STRAD, and RBM4 were examined in KYSE150 and KYSE450 cells with stable depletion (**a**) or overexpression (**b**) of RBM4. **c** Immunoprecipitation assay was performed in KYSE150 cells expressing Flag-RBM4, HA-LKB1 or Flag-LKB1, HA-RBM4 respectively, and the protein complex precipitated by Flag-M2 agarose beads were analyzed through western blotting. **d**, **e** Immunoprecipitation assay was carried out in KYSE150 cells expressing a fixed amount of Flag-MO25 and increased amount of RBM4 (**d**), or elevated amount of siRBM4 (e, left), or a fixed amount of Flag-LKB1 with increased amount of siRBM4 (**e**, right) respectively after 8-h PS341 (10 μM) treatment. The protein complexes were precipitated with anti-Flag followed by western blotting analysis. **f** Immunoprecipitation assay was performed in KYSE150 cells expressing HA-LKB1 and Flag-RBM4, or Flag-RBM4(1-177), Flag-RBM4(78-364), Flag-RBM4(178-364) in the presence of PS341 (10 μM). The Flag-tagged precipitated-complexes were analyzed with a specific HA or Flag antibody. Asterisks indicate non-specific bands. **g** Immunoprecipitation assay was carried out in KYSE150 cells expressing Flag-RBM4 and HA-LKB1, or HA-LKB1(1-243), HA-LKB1(1-317), HA-LKB1(Δ146-186), HA-LKB1(88-243), HA-LKB1(88-433) in the presence of PS341 (10 μM). The Flag-tagged precipitated-complexes were analyzed using western blotting. **h** The levels of LKB1 and RBM4 were examined in the cytoplasm and nucleus of KYSE150 cells expressing HA-RBM4 following PS341 (10 μM) treatment for 8 h. SE, short exposure; LE, long exposure. **i** Confocal immunofluorescence microscopy was utilized to examine the localization of Flag-RBM4 and HA-LKB1 in Cos7 cells. Scale bar = 10 μm

We next investigated how RBM4 represses the expression of LKB1. Different from the pronounced increase of protein level, the mRNA level of LKB1 was not significantly affected by RBM4 depletion (Supplementary Fig. 3c). Since RBM4 has been known as a splicing factor, we designed RT-qPCR primers specifically targeting two isoforms of LKB1 to analyze whether RBM4 inhibited LKB1 expression by regulating its alternative splicing. The data showed that knockdown or overexpression of RBM4 did not significantly impact the mRNA level of LKB1 isoform 1, which encodes the canonical LKB1, or LKB1 isoform 2, the short isoform, in ESCC cells, suggesting that RBM4 could not regulate alternative splicing of LKB1 mRNA (Supplementary Fig. 3d). According to previous reports, MO25 and STRAD (e.g. functions as an anchor for LKB1) bind to LKB1 to form a heterotrimeric complex, thereby promoting the catalytic activity and stability of LKB1.^58,59^ We thus examined whether RBM4 influenced the level of MO25 or STRAD. Interestingly, RBM4 did not impact the level of MO25 and STRAD in both KYSE150 and KYSE450 cells (Fig. 4a, b). Nevertheless, RBM4 interacted with both exogenously expressed and endogenous LKB1 in immunoprecipitation assays (Fig. 4c and Supplementary Fig. 3e). However, RBM4 did not interact with STRAD (Supplementary Fig. 3f), suggesting that RBM4 might interact with LKB1 to disrupt the LKB1/MO25/STRAD heterotrimeric complex, thus altering the conformation and stability of LKB1 protein. To test this hypothesis, we co-transfected a fixed amount of MO25 with increased amounts of RBM4 respectively, and performed immunoprecipitation assay. As expected, the interaction between RBM4 and LKB1 disrupted the interaction of MO25 and LKB1 in a dose-dependent manner, as less LKB1 protein was precipitated by anti-Flag antibody upon increased ectopic expression of RBM4 (Fig. 4d). Conversely, the interaction between MO25 and LKB1 or STRAD was dose dependently elevated with gradual knockdown of RBM4 with siRNAs in an immunoprecipitation assay with anti-Flag-MO25 (Fig. 4e). Similar results were also obtained in another immunoprecipitation assay with anti-Flag-LKB1 (Fig. 4e). To determine which domain of RBM4 is responsible for the interaction with LKB1, we constructed a series of RBM4 truncated mutants. As shown in Fig. 4f, the RBM4 mutant lacking 1^st^ to 177^th^ or even 1^st^ to 77^th^ amino acids failed to interact with LKB1, whereas the mutant lacking 178^st^ to 364^th^ could still bind to LKB1, indicating that RBM4 binds to LKB1 through the RNA recognition motifs containing N-terminus. We further examined which region of LKB1 is required for interacting with RBM4. Among a series of truncated LKB1, only the mutants lacking the N-terminal tail (1^st^ to 87^th^ amino acids) lost the capacity to associate with RBM4 (Fig. 4g). It has been previously reported that the N-terminal lob of LKB1 interacted with STRAD,^60^ indicating that the binding of RBM4 to LKB1 might be able to abolish the interaction between LKB1 and STRAD. Considering that STRAD was capable of anchoring LKB1 in the cytoplasm,^58,61^ we next ask whether the high expression level of RBM4 affects the nucleocytoplasmic shuttling of LKB1. Nuclear and cytoplasmic fractionation experiments were performed to relatively quantify and analyze the cytoplasmic versus the nuclear LKB1. We found that the expression of RBM4 promoted the accumulation of LKB1 in the nucleus in KYSE150 and KYSE30 cells (Fig. 4h and Supplementary Fig. 3g). In line with this, RBM4 evidently stimulated the translocation of LKB1 to the nucleus in a confocal immunofluorescence assay (Fig. 4i and Supplementary Fig. 3h). Taken together, our data suggest that RBM4 competitively binds to LKB1 to disrupt the formation of LKB1/STRAD/MO25 heterotrimeric complex when overexpressed in cells, thereby promoting the re-localization of LKB1 from the cytoplasm to the nucleus.

### RBM4 recruits E3 ligase TRIM26 to promote LKB1 degradation in nucleus

**Fig. 5:**
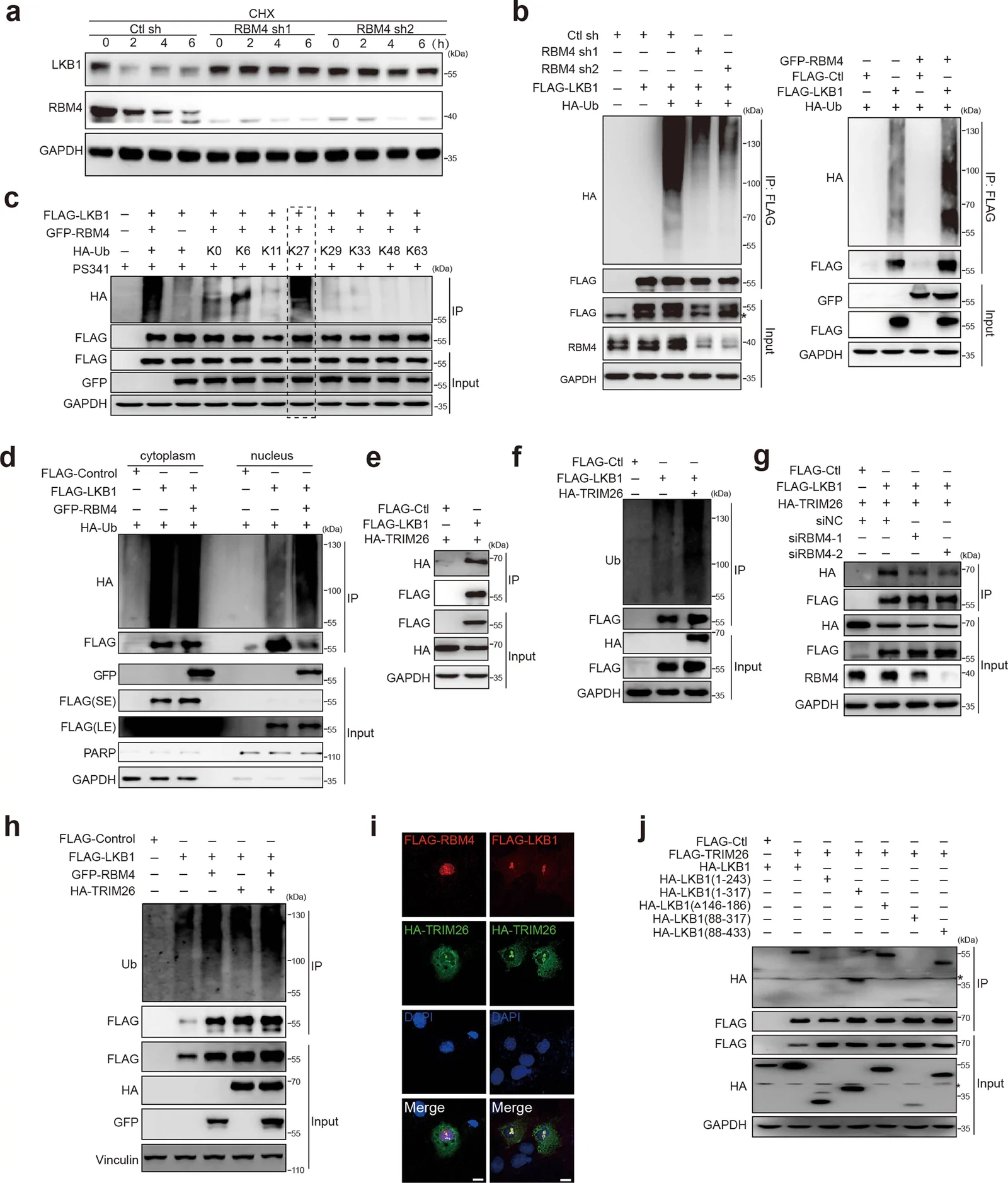
RBM4 recruits E3 ligase TRIM26 to promote LKB1 degradation in nucleus. **a** KYSE150 cells with RBM4 stably knockdown were treated with 50 μg/mL cycloheximide (CHX) for the indicated time course. The protein levels of LKB1 and RBM4 were detected by a western blot assay. **b** Immunoprecipitation assay was performed in RBM4-depleted KYSE150 cells co-expressing Flag-LKB1 following PS341 (10 μM) treatment for 8 h to examine the ubiquitination of LKB1. The protein complexes were precipitated by anti-Flag M2 resin and analyzed with the indicated antibodies. Asterisks indicate non-specific bands. **c** Immunoprecipitation assay was carried out in cells expressing Flag-LKB1 and GFP-RBM4, with different HA-Ub vectors containing distinct lysine mutations in the presence of PS341 (10 μM). The protein complexes were precipitated by anti-Flag M2 resin and analyzed by a western blot assay. **d** KYSE150 cells co-transfected with the indicated vectors expressing Flag-LKB1, GFP-RBM4 or HA-Ub in the presence of PS341 (10 μM) were subjected to nucleocytoplasmic fractionation followed by a co-immunoprecipitation assay with anti-Flag M2 resin to analyze the ubiquitination of LKB1 in the cytoplasm and nucleus respectively. SE, short exposure; LE, long exposure. **e** The interaction between LKB1 and TRIM26 was determined by an immunoprecipitation assay with anti-Flag antibody following PS341 (10 μM) treatment for 8 h. **f** Immunoprecipitation assay was carried out to determine the Flag-LKB1-bound poly-ubiquitin with or without HA-TRIM26 overexpression following PS341 (10 μM) treatment for 8 h. **g** Immunoprecipitation assay was performed in RBM4-depleted or control cells after PS341 (10 μM) treatment for 8 h to assess the interaction between LKB1 and TRIM26. **h** Immunoprecipitation assay was carried out to investigate the effect of RBM4 and TRIM26 on the ubiquitination of LKB1 in the presence of PS341 (10 μM). **i** Confocal immunofluorescence microscopy was used to examine the localization of Flag-RBM4 or Flag-LKB1 with HA-TRIM26 in HeLa cells. Scale bar = 10 μm. **j** Immunoprecipitation assay was performed in KYSE150 cells expressing Flag-TRIM26 and HA-LKB1, or HA-LKB1(1-243), HA-LKB1(1-317), HA-LKB1(Δ146-186), HA-LKB1(88-317), HA-LKB1(88-433) in the presence of PS341 (10 μM). The Flag-tagged precipitated-complexes were analyzed by western blotting. Asterisks indicate non-specific bands

Subsequently, we sought to investigate the consequence of RBM4-promoted nuclear translocation of LKB1. Consistent with the previous study that the nuclear translocation is responsible for LKB1 degradation,^58,62^ we found that depletion of RBM4 notably stabilized the level of LKB1 as compared to control (Fig. 5a). Then we examined protein levels of LKB1 in RBM4-depleted ESCC cells in the presence or absence of proteasome inhibitor Bortezomib/PS341. The results showed that PS341 promoted LKB1 protein accumulation and at least partially abolished the increase of LKB1 caused by RBM4 depletion, suggesting that the regulation of LKB1 by RBM4 relies on ubiquitin-proteasome pathway (Supplementary Fig. 4a). In addition, knockdown of RBM4 inhibited the ubiquitination of LKB1, whereas overexpression of RBM4 promoted LKB1 ubiquitination in the presence of PS341 (Fig. 5b). Specifically, RBM4 stimulated the K27-linked ubiquitination of LKB1, however, other lysine-linked ubiquitination was not significantly influenced by RBM4 (Fig. 5c). Consistent with the results in Fig. 4h, i, instead of in the cytoplasm, RBM4 promoted the ubiquitination of LKB1 in the nucleus (Fig. 5d). Altogether, our data suggested that RBM4 might promote the ubiquitination of LKB1 in the nucleus, thereby stimulating its subsequent degradation.

We performed an immunoprecipitation assay coupled with Mass Spectrometry (IP-MS) using RBM4 as a bait, and found that twelve E3 ligases interacted with RBM4. We next sought to determine whether any of these E3 ligases is responsible for the RBM4-mediated LKB1 degradation. We found that knockdown of TRIM26, but not other examined E3 ligases, could evidently elevate the protein of LKB1 (Supplementary Fig. 4b). Additional validation showed that depletion of TRIM26 promoted the protein level of LKB1, whereas overexpression of TRIM26 strikingly reduced the expression of LKB1 (Supplementary Fig. 4c). Nevertheless, RBM4 knockdown did not induce the reduction of TRIM26, and vice versa (Supplementary Fig. 4d). Moreover, the decreased LKB1 level caused by ectopic expression of RBM4 could be reversed by the depletion of TRIM26 (Supplementary Fig. 4e), indicating that the existence of TRIM26 is required for RBM4 to control LKB1 expression. Furthermore, immunoprecipitation assays revealed that TRIM26 could either interact with RBM4 or LKB1 (Fig. 5e and Supplementary Fig. 4f, g), but the level of RBM4 was not significantly influenced by TRIM26 (Supplementary Fig. 4c). Thus, we speculated that RBM4 might function as a recruiter in targeting LKB1 by TRIM26 to promote LKB1 ubiquitination. As expected, ectopic expression of TRIM26 enhanced the amount of ubiquitin pulled down by Flag-LKB1 (Fig. 5f). The interaction between TRIM26 and LKB1 was obviously disrupted by the knockdown of RBM4 (Fig. 5g). Moreover, TRIM26 promoted the ubiquitination of LKB1, which could be further enhanced by overexpression of RBM4 (Fig. 5h). Meanwhile, RBM4 and LKB1 colocalized with TRIM26 respectively in the nucleus following PS341 treatment (Fig. 5i). We further investigated which region of LKB1 could interact with TRIM26, and found that TRIM26 specifically bound to the region containing 243th–317th amino acids of LKB1 (Fig. 5j). Taken together, our data suggested that RBM4 recruited LKB1 to E3 ligase TRIM26 in the nucleus, thus promoting the ubiquitination of LKB1 and its subsequent nuclear degradation.

### Glutamine metabolism elevated by RBM4-LKB1 axis promotes ESCC cells survival

**Fig. 6:**
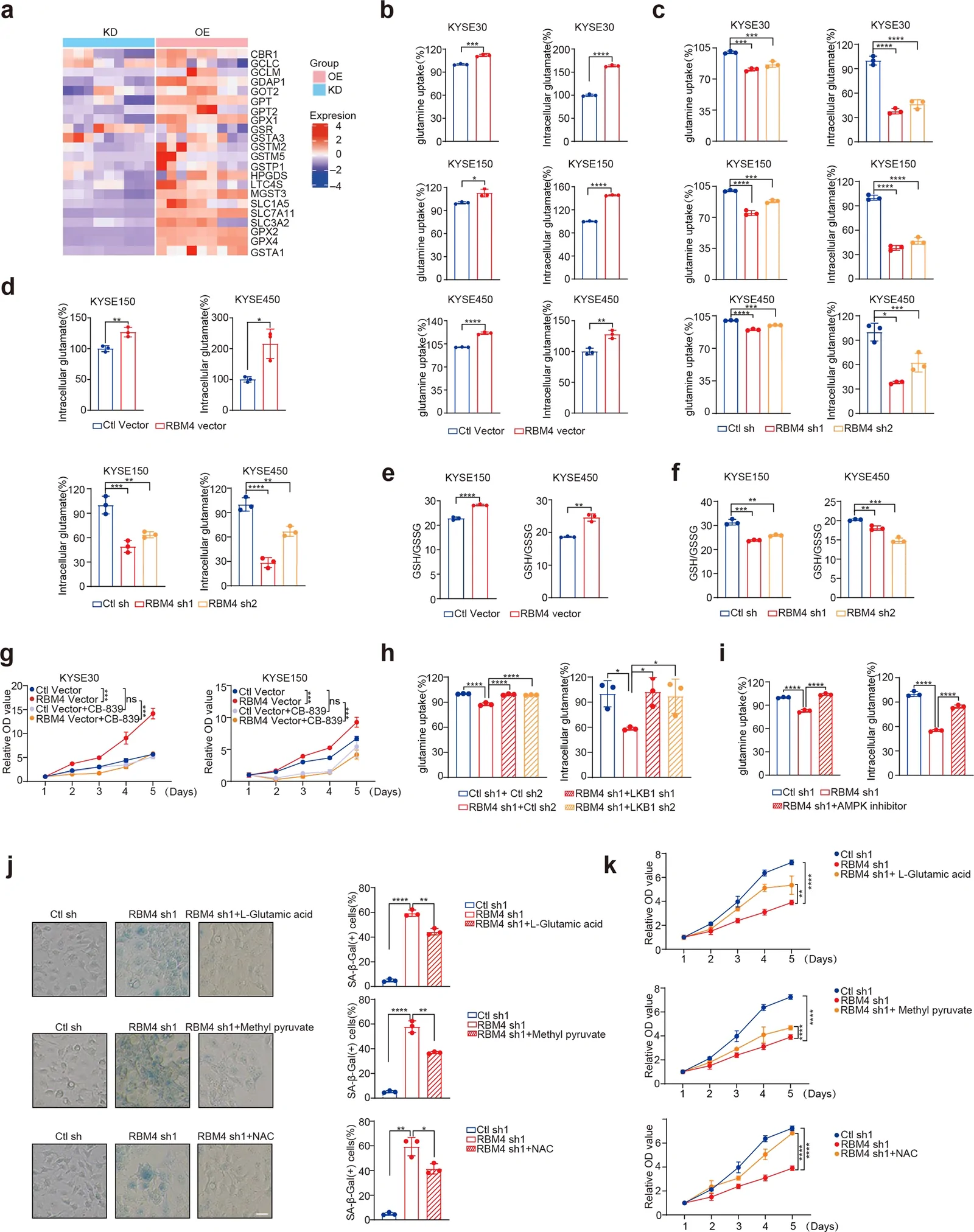
Glutamine metabolism was elevated by RBM4-LKB1 axis to promote ESCC cells survival. **a** Heatmap analysis of metabolism-related genes upon RBM4 depletion or overexpression in KYSE150 cells. **b**, **c** Glutamine consumption and glutamate levels were examined in KYSE30, KYSE150 and KYSE450 cells with stable overexpression of RBM4 (**b**) or depletion of RBM4 (**c**). Three experiments were performed and mean ± SD was plotted. *P*-values were determined by Student’s *t*-test (**b**) or One-way ANOVA with Dunnett multiple comparisons (**c**). **d** The glutamate production was examined in KYSE150 and KYSE450 cells with stable overexpression of RBM4 or depletion of RBM4 while glutamine deprivation. Three experiments were performed and mean ± SD was plotted. *P*-values were determined by Student’s *t*-test or One-way ANOVA with Dunnett multiple comparisons. **e**, **f** KYSE150 and KYSE450 cells with stable overexpression of RBM4 (**e**) or depletion of RBM4 (**f**) were subjected to measure the reduced GSH/ oxidized GSSG ratio calculated by GSH/GSSG = [Total GSH-(2 × GSSG)]/GSSG. Three experiments were performed and mean ± SD was plotted (*P*-values were determined by One-way ANOVA with Dunnett multiple comparisons or Student’s *t*-test). **g** The growth curve of RBM4-overexpressed KYSE30, and KYSE150 cells with or without CB-839 treatment was measured by CCK8 assay. *P-*values were determined by two-way repeated measures ANOVA. **h** Glutamine consumption and glutamate levels of RBM4-depleted KYSE150 cells in the presence or absence of LKB1 were examined. Three experiments were performed with mean ± SD plotted (*P*-values were determined by One-way ANOVA with Dunnett multiple comparisons). **i** Glutamine consumption and glutamate levels of RBM4-depleted KYSE150 cells in the presence or absence of AMPK inhibitor compound C (5 μM) were examined. Three experiments were performed with mean ± SD plotted (*P*-values were determined by One-way ANOVA with Dunnett multiple comparisons). **j** β-gal staining of KYSE150 cells with stable knockdown of RBM4 in the presence or absence of glutamic acid (0.5 mM), methyl pyruvate (10 mM) and NAC (3 mM). Three experiments were carried out with mean ± SD of β-gal positive cells plotted (*P*-values were determined by One-way ANOVA with Dunnett multiple comparisons). Scale bar = 25 μm. **k** The growth curve of RBM4-depleted KYSE150 cells with or without addition of glutamic acid, methyl pyruvate, and NAC was measured by CCK8 assay. *P*-values were determined by two-way repeated measures ANOVA. *P < 0.05, **P < 0.01, ***P < 0.001, ****P < 0.0001

Previous studies have demonstrated that loss of LKB1 elevated glutamine uptake and flux in proliferative cells for biosynthesis and bioenergetic needs.^25,63^ Due to the potential preference of ESCC to glutamine metabolism and the dependency of cancer cells on glutamine supply,^5,10^ we speculated whether glutamine metabolism is involved in the tumor-promoting function of RBM4-LKB1 axis. We examined the transcriptional levels of a series of genes associated with glutaminolysis pathway, which have been reported to be downregulated by LKB1 deficiency,^64^ in ESCC cells with overexpression or depletion of RBM4. The majority of glutaminolysis-related genes were markedly increased upon RBM4 expression, whereas the levels were decreased upon RBM4 knockdown as compared to the control group (Fig. 6a). In addition, overexpression of RBM4 enhanced the glutamine consumption and the concomitant glutamate production in different ESCC cell lines (Fig. 6b), while depletion of RBM4 suppressed the cellular glutamine consumption and glutamate production (Fig. 6c), suggesting that RBM4 might promote glutamine utilization of ESCC cells. While in the context of glutamine-deprived media, RBM4 overexpression still promoted the glutamate production, and vice versa (Fig. 6d), implying RBM4 might be implicated in the regulation of glutamate biosynthesis. Glutathione (GSH), an anti-oxidative ROS scavenger, is generated by the reaction of L-glutamate with cysteine and glycine from glutamine.^65^ In concert, we found a higher GSH amount in RBM4-overexpressed ESCC cells, and vice versa (Fig. 6e, f). We next investigated whether the glutamine metabolism changes were involved in the oncogenic property of RBM4 in ESCC. We therefore used CB-839, an allosteric inhibitor of glutaminase (GLS), to block the conversion from glutamine to glutamate in ESCC cells with stable overexpression of RBM4 or control. As expected, such treatment significantly abolished RBM4 overexpression-induced accelerated growth of ESCC cells (Fig. 6g). Collectively, our data suggest that high RBM4 expression enhances glutamine utilization to support cell proliferation in ESCC.

Consistent with this, we verified that LKB1-depletion was capable of rescuing the RBM4 depletion-induced inhibition of the glutamine consumption, and glutamate production (Fig. 6h), whereas in RBM4 stably overexpressing ESCC cells, re-expression of LKB1 overturned the upregulated consumption of glutamine and the high rate of glutamate production (Supplementary Fig. 5a). AMPK, the canonical substrate of LKB1, is a well-known metabolic hub that was previous reported to negatively affect glutaminolysis via its kinase activity.^23,66^ To assess whether AMPK is involved in the RBM4-regulated glutamine metabolism, we measured the glutamine flux by the addition of Compound C, an ATP-competitive AMPK inhibitor, in RBM4-knocked down ESCC cells. In agreement to the reversed effect of LKB1 reduction, such inhibition of AMPK antagonized the reduced glutamine uptake and glutamine production led by RBM4-knockdown (Fig. 6i). Meanwhile, we performed qRT-PCR assays to measure changes of glutaminolysis-related genes in RBM4-depleted cells while in the absence of LKB1, and found the decrease of plenty of genes induced by RBM4 deletion were abolished by LKB1 knockdown (Supplementary Fig. 5b), suggesting LKB1 degradation may mediate the function of RBM4 in promoting glutamine metabolism. Otherwise, a few glutaminolysis-associated genes, such as GPT2, in RBM4-knockdown ESCC cells cannot be fully rescued by LKB1 deletion, implying the possibility that RBM4 might work through other mechanisms besides LKB1.

We have shown that RBM4 reduction restricted ESCC cellular consumption of glutamine, which is a versatile nutrient that participates in biomass, including purine and pyrimidine synthesis, energy production and redox homeostasis.^67^ Therefore, we examined the tentative effect of altered glutamine metabolism in RBM4-reduction induced cellular senescence and proliferative arrest. Additional glutamic acid was supplemented in the culture medium of RBM4-depleted KYSE150 and KYSE450 cells for 48 h. The application of glutamic acid partly reversed the number of senescent cells resulted from RBM4 depletion (Fig. 6j and Supplementary Fig. 5c). In addition, methyl pyruvate, an energy source, could partially overturn RBM4 depletion-induced cellular senescence (Fig. 6j and Supplementary Fig. 5c). As depicted in Fig. 6f, RBM4 depletion leaded to GSH production deficiency, which is a crucial cause in ROS accumulation. Indeed, treatment of RBM4 depleted ESCC cells for 48 h with NAC, the ROS scavenger, could also trigger a pronounced decrease in the population of senescent cells induced by RBM4 knockdown (Fig. 6j and Supplementary Fig. 5c). In line with these observations, no matter for glutamic acid remedy or methyl pyruvate treatment or NAC application, the suppression of KYSE150 and KYSE450 cell growth induced by RBM4 knockdown could be partially rescued (Fig. 6k and Supplementary Fig. 5d). Our above-described data indicate that glutamine metabolism was elevated by RBM4-LKB1 axis to evade cellular senescence and support ESCC cells survival. As previous reports claimed that glutamine metabolism was necessary for senescent cells survival or senescence bypass,^37,68^ we speculated that the impaired glutamine metabolism might hinder progression of RBM4-depleted senescent cells. Conversely, ESCC cells with high expression of RBM4 and its concomitant inhibition of LKB1 activity have a stronger ability to fuel glutamine, which may assist stressed cells to escape from pre-mature aging and maintain a high proliferative rate.

### The RBM4 high/LKB1 low expression endows ESCC metabolic vulnerability

**Fig. 7:**
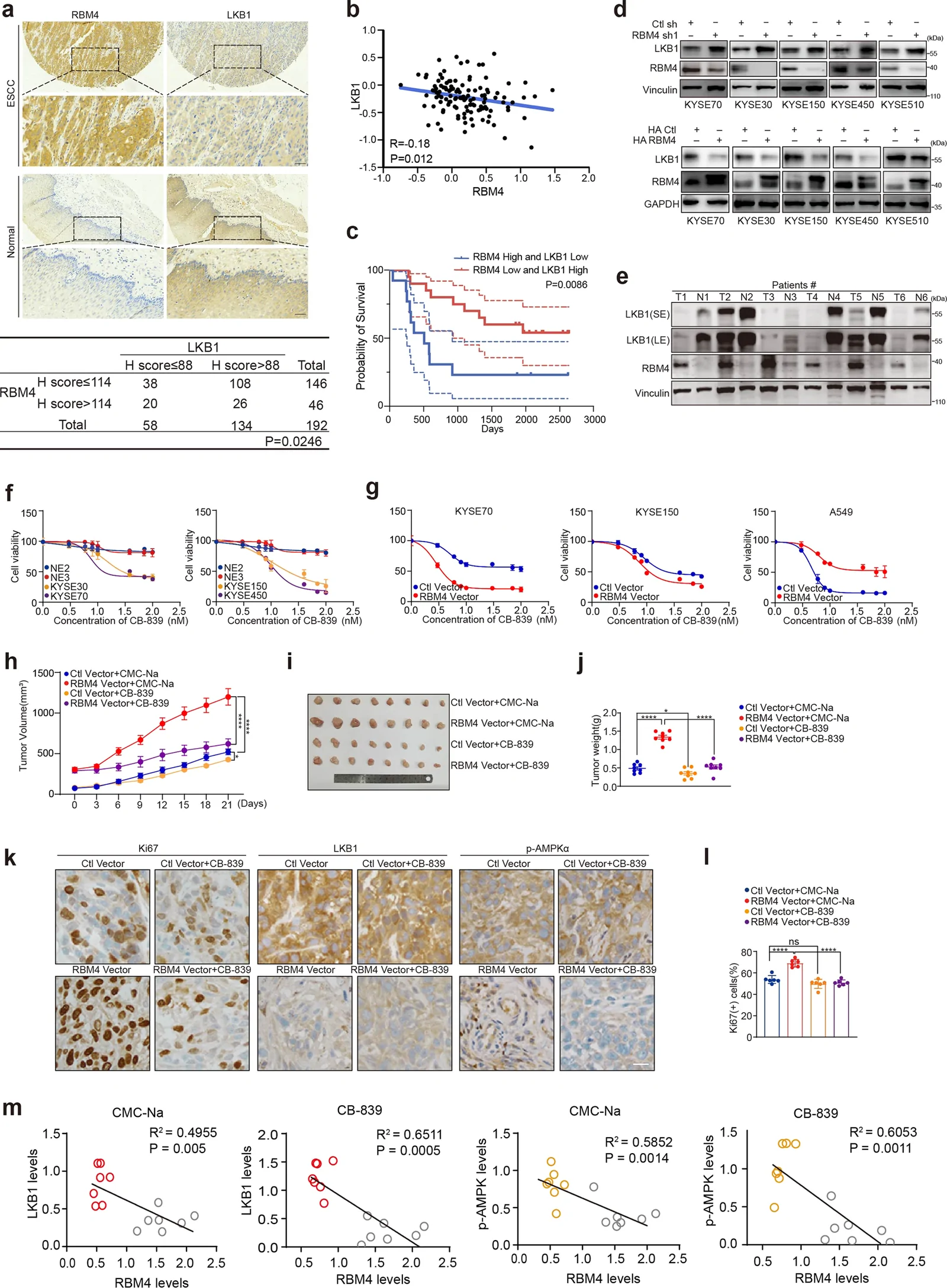
RBM4 endows ESCC metabolic vulnerability. **a** The tissue microarray of ESCC patients was subjected to IHC staining for the specific RBM4 and LKB1 antibodies. Representative images were acquired with ×10 and ×40 objectives (scale bar = 50 μm). The results were analyzed by the pathologist who provided a value ranging from 0 to 3 for each sample. “0” indicates negative staining; “1” means weak positive staining; “2” denotes moderately positive staining and “3” indicates strongly positive staining. H-score is obtained by the formula: 3 × percentage of strongly staining + 2 × percentage of moderately staining + percentage of weakly staining. The correlation between RBM4 and LKB1 expression in ESCC and the corresponding normal tissues was calculated by Pearson’s chi-square test. **b** Correlation of RBM4 and LKB1 levels were analyzed using data obtained from mass spectrometry-based proteomic profiling of ESCC tumors and adjacent non-tumor tissues (PXD021701). (*P* = 0.012 by Pearson’s chi-square test). **c** Kaplan–Meier curve showing overall survival of ESCC patients (PXD021701) with high RBM4 expression and low LKB1 expression or low RBM4 expression and high LKB1 expression (*P* = 0.0086 by log-rank test, dotted red/blue indicates the upper and lower confidence limits.). **d** Protein levels of LKB1 and RBM4 were determined in RBM4-depleted or overexpression ESCC cells, including KYSE30, KYSE70, KYSE150, KYSE450, and KYSE510 cells. **e** Protein levels of LKB1 and RBM4 in fresh-frozen tumor and the corresponding normal tissue specimens from ESCC patients were examined by western blot assay. “*N*” stands for the adjacent non-tumor tissues, “T” represents ESCC tissues. SE, short exposure; LE, long exposure. **f** Dose-response curves of cell viability in normal esophageal epithelial cells (NE2 and NE3) and distinct ESCC cells (KYSE30, KYSE70, KYSE150, and KYSE450) treated with CB-839 (*n* = 3; mean of three technical replicates from a representative experiment was shown in the graph) for 72 h. **g** Dose-response curves of cell viability in RBM4-overexpression cells (KYSE70, KYSE150, and A549) treated with CB-839 (*n* = 3; mean of three technical replicates from a representative experiment was shown in the graph) for 72 h. **h–j** Nude mice were subcutaneously injected with 5 × 10^6^ KYSE30 cells with stable overexpression of RBM4 or control respectively. When the tumor reached 50–250 mm^3^, the animals were randomized to the respective treatment groups. CB-839 (200 mpk) was administered by oral gavage twice a day, tumor volumes were measured every 3 days (**h**). After 21 days of treatment, the mice were sacrificed to remove the xenografted tumors as shown in (**i**), and the tumors weights were measured (**j**). *n* = 7, error bars indicate mean ± SEM. *P-*values were determined by two-way repeated measures ANOVA in H or one-way ANOVA with Dunnett multiple comparison in J. *P < 0.05, ****P < 0.0001. **k**, **l** After 21 days of oral administration of CB-839 or vehicle (0.5% CMC-Na), the nude mice bearing RBM4-overexpressing or empty vector-expressing xenografts were sacrificed to remove tumors for immunohistochemistry analysis of the proliferation marker Ki67, LKB1 and phosphorylation of AMPK (**k**). Scale bar = 50 μm. Quantification of Ki67 positive cells after IHC analysis was performed using image J software and plotted as mean ± SD with *P*-value in ANOVA test (**l**). ****P < 0.0001. **m** 21-days after oral administration of CB-839 or vehicle (0.5% CMC-Na), the nude mice bearing RBM4-overexpressing or empty vector xenografts were sacrificed to remove tumors for western blot and gray value analysis of RBM4, LKB1 and phosphorylation of AMPK. The correlation between the levels of RBM4 and LKB1/p-AMPK was calculated by Pearson correlation coefficient calculation and plotted. Gray circle represents RBM4-overexpressed samples, red/orange circle represents control vector samples. R, Pearson correlation coefficient; *P*-value, two-tailed *P*-value of the Pearson correlation

In light with these findings, we next wanted to clarify the clinical relevance between RBM4 and LKB1. To this end, we measured the protein expression of RBM4 and LKB1 on human ESCC tissue microarrays containing 192 tumor tissue and adjacent normal tissue samples by immunohistochemistry staining. According to the statistical analysis based on a receiver operating characteristic plot, RBM4 expression is negatively correlated to the level of LKB1 in ESCC tissues and the corresponding normal tissues (Fig. 7a). In addition, we also analyzed the data obtained from mass spectrometry-based proteomic profiling of ESCC tumors and adjacent non-tumor tissues,^69^ and found that RBM4 was increased, whereas LKB1 was decreased in ESCC samples (Supplementary Fig. 6a), further Spearman’s rank correlation analysis proved a significant and negative correlation between RBM4 and LKB1 (Fig. 7b). Consistently, ESCC patients with the low RBM4 expression and high LKB1 expression had significantly better survival than patients with a high RBM4 expression and low LKB1 expression (Fig. 7c). We subsequently applied different ESCC cells bearing distinct genetic background (e.g.P53, EGFR, CDKN2A, NFE2L2 status) to engineer RBM4 expression by infecting with overexpression or knockdown lentivirus. As illustrated in Fig. 7d, depletion of RBM4 improved the level of LKB1, whereas overexpression of RBM4 reduced the LKB1 expression irrespectively of genetic background of the examined ESCC cells, further proving that RBM4 may participate in the regulation of ESCC progression through reversely regulating LKB1. We also collected fresh-frozen tumor and the corresponding normal tissue specimens from ESCC patients and examined the protein levels of RBM4 and LKB1. As demonstrated in new Fig. 7e, tumor samples with high expression of RBM4 exhibited decreased LKB1 levels. Consistently, we found that the protein levels of LKB1 and its downstream target P27 were markedly reduced in a panel of ESCC cells compared to normal esophageal epithelial cells (NE2 and NE3), which is opposite to the high expression of RBM4 in ESCC cells (Supplementary Fig. 6b).

We further observed that the normal esophageal epithelial cells NE2 exhibited inferior ability of glutamine uptake as compared to KYSE30, KYSE70, KYSE150 and KYSE450 ESCC cells (Supplementary Fig. 6c). The rate of glutamate production was heightened in ESCC cells versus NE2 in the condition of glutamine abundance or deprivation (Supplementary Fig. 6c). However, A549 lung adenocarcinomas cells, which are null for LKB1, were not prone to utilize glutamine whilst being reconstituted with RBM4 expression (Supplementary Fig. 6d), confirming the association between RBM4-LKB1 axis and glutamine utilization in ESCC cells as observed in above findings. The application of CB-839 could markedly halt the enhanced RBM4-induced acceleration of glutamine consumption and glutamate secretion in ESCC cells (Supplementary Fig. 6e), therefore, we sought to investigate whether RBM4-induced glutamine preference could endow ESCC cells with intrinsic vulnerability, sensitizing ESCC cells to the glutaminase inhibitor CB-839, which is similar to the consequence of LKB1 deficiency in lung cancer. To test this hypothesis, the half-maximal inhibitory concentration (IC50) for CB-839 was assessed in NE2 and NE3 cells with low RBM4 expression and ESCC cells with high RBM expression. As shown in Fig. 7f, ESCC cells were indeed more sensitive to CB-839 than NE2 and NE3 cells. In addition, we found that ectopic expression of RBM4 sensitized ESCC cells to CB-839 as the IC50 was notably reduced by RBM4-expression, but this phenomenon was not noticed in LKB1-null A549 cells (Fig. 7g). The addition of glutamic acid was sufficient to ameliorate the growth inhibition led by CB-839 in RBM4-overexpressed ESCC cells (Supplementary Fig. 6f), indicating the glutamine-addiction of ESCC with high RBM4 expression. To validate the phenomenon in vivo study, KYSE30 xenografts tumors expressing RBM4 or control vector were established in nude mice and administrated by CB-839 (200 mpk) or vehicle (0.5% CMC-Na) twice a day. Tumor volumes were measured every 3 days until mice were sacrificed and tumor dissected. The results showed that the growth rate and size/weight of RBM4-high tumors were more markedly inhibited than control tumor after 21 days CB-839 administration (Fig. 7h–j). Immunohistochemical analysis was performed on paraffin sections from xenografts, which showed that the percentage of proliferation marker Ki67 positive cells were significantly decreased in RBM4-overexpressing xenografts treated with CB-839 compared to drug vehicle (Fig. 7k, l). Meanwhile, immunochemistry staining and immunoblot assays both proved that the protein level of LKB1 and the phosphorylation of AMPK was reduced in xenografts derived from RBM4-overexpressing ESCC cells (Fig. 7m and Supplementary Fig. 6g, h), which showed a greater sensitivity to glutaminase inhibitor, CB-839, suggesting that RBM4-LKB1-AMPK axis is involved in the glutamine dependency of ESCC cells. Taken together, our data suggest that the RBM4^high^/ LKB1^low^ expression defines glutamine metabolic reprogramming and confer increased sensitivity of ESCC cells to glutaminase inhibition, providing a novel potential target for ESCC therapeutics.

## Discussion

**Fig. 8:**
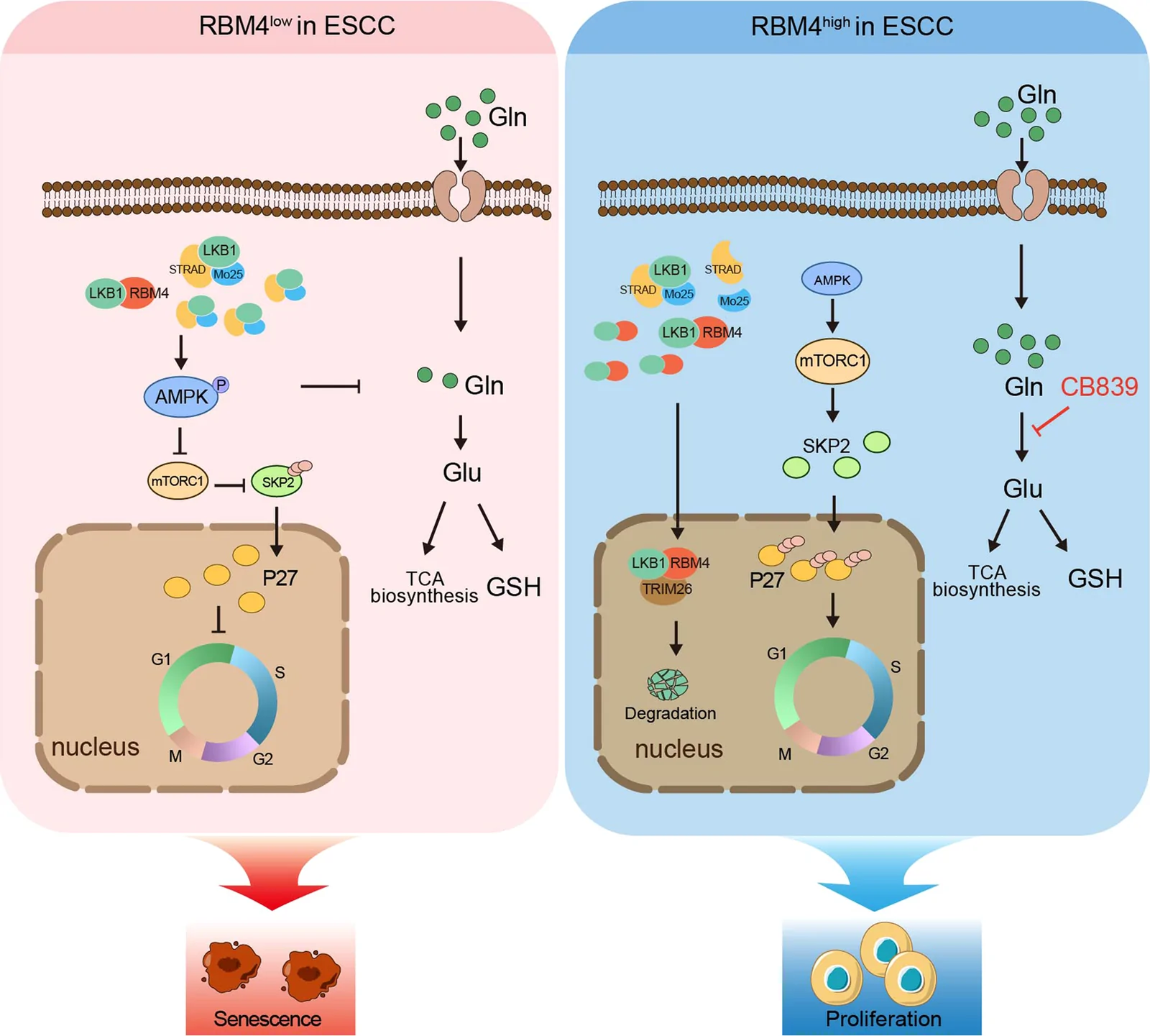
Schematic of how RBM4 dictates ESCC cells fate from senescence to Glutamine-addiction survival through regulating LKB1-AMPK-P27 pathway. Mechanistically, RBM4 competitively bound to LKB1 to disrupt the formation of LKB1/STRAD/MO25 heterotrimeric complex and recruited the E3 ligase TRIM26 to LKB1, promoting the ubiquitination of LKB1 and its subsequent degradation, thereby conferring the enhanced sensitivity of glutaminase inhibitor CB-839 to RBM4-high ESCC. Conversely, RBM4 depletion stabilized LKB1 to activate p-AMPK, thus inhibiting mTOR activity and inducing P27-dependent senescence. The illustration of proliferative cells used elements from Servier Medical Art (https://smart.servier.com/) by Servier under a Creative Commons Attribution 3.0 Unported License

Previously, RBM4 was reported to inhibit cancer progression of multiple types of human cancers, including lung, breast, gastric, colorectal, and hepatocellular cancer.^39,41,42,46,47^ For example, RBM4 regulates the splicing of Bcl-x and TEAD4 to suppress lung cancer progression^39,40^; SRPK1-RBM4 network contributes to tumorigenesis via altered sensitivity to apoptotic signals in breast cancer cells.^42^ However, the role of RBM4 in ESCC has not been characterized yet. Surprisingly, we revealed that RBM4 is highly expressed in ESCC and promotes Glutamine-dependent proliferation. Depletion of RBM4 induces P27-dependent cancer cell senescence and inhibits ESCC progression, which could be reversed by glutamic acid. Our data showed that RBM4 overexpression, to a certain extent, prevents various stressors-induced senescence. Mechanistically, RBM4 disrupts the LKB1/MO25/STRAD complex and recruits TRIM26 to promote LKB1 degradation, rendering ESCC cells susceptible to glutaminase inhibitor, CB-839, and offering a novel approach for ESCC targeted therapy (Fig. 8). In line with the tumor-promoting function of RBM4 in ESCC, analysis of the RNA-seq data from TCGA and GEO dataset revealed that RBM4 is highly expressed in ESCC, while RBM4 is often downregulated in other cancer cell lines, such as colorectal and liver cancer.^41,46,70^ We constructed colorectal and liver cancer cell lines with stably overexpressed or depleted RBM4. We found LKB1 protein level was not manipulated by RBM4 expression (Supplementary Fig. 6i). Thus, we speculated that distinct genetic backgrounds in these cell lines led to the interruption of RBM4-mediated regulation of LKB1 stability. Moreover, RBM4 reduction also elevated the activity of senescence marker, SA-β-gal, in colorectal and liver cancer cell lines (Supplementary Fig. 6j), but not increased p27 expression (Supplementary Fig. 6i), suggesting the underlying mechanism is different from that in ESCC cells. We proposed that these findings might result from tissue specificity and tumor genotype, similar to the distinct roles of PTEN in different cancers, but the underlying specific molecular cues remain to be further explored.^71^The roles of senescence in tumor are paradoxical and depends on specific cellular or extracellular circumstances.^72–75^ As a barrier for tumorigenesis, cellular senescence was known to be circumvented by cancer cells rewiring their metabolism.^76–79^ Since RBM4 depletion not only induced cellular senescence, but also impeded glutamine utilization by upregulating LKB1 levels in ESCC, we assumed that it’s a combination of cell cycle exit and hampered glutamine metabolism results in depleted RBM4-induced ESCC suppression.

The data presented herein describe a non-canonical role of RBM4 in dictating the activity of LKB1, an essential metabolic regulator, at the protein level. Such role is supported by the observations that RBM4 could not impact LKB1 mRNA level, but dramatically decrease the steady state level of LKB1. Prior studies have revealed that LKB1 is anchored in the cytosol by STE20-related pseudokinase (STRAD), and the interaction is stabilized by the scaffolding protein MO25.^58,59,62^ The catalytic activity of LKB1 is enhanced over a hundred-fold by a conformational change in the LKB1/STRAD/MO25 complex.^60,80^ However, how the active complex is disrupted, and the detailed mechanisms underlie the degradation of LKB1 is obscure. Although it has been previously reported that CHIP interacts with LKB1 and regulates the proteosome-mediated degradation of LKB1,^81^ we didn’t discover the association between CHIP and LKB1 in our cell models. In this study, we found that overexpressed RBM4 hampered the formation of the heterotrimeric complex by interacting with the LKB1 N-terminal lobe (the residues 1th–88th aa), which is consistent with previous reports that mutation of Arg 74 on LKB1 abrogated its binding to STRAD and decreased the complex catalytic function.^60^ We revealed that RBM4 promoted the translocation of LKB1 from cell cytosol to nucleus and fortified the attachment of E3 ligase TRIM26 to the 243th–317th aa region of LKB1. TRIM26 is a member of the Tripartite motif (TRIM) protein family, which is implicated in innate immunity, DNA damage response, tumor progression.^82,83^ TRIM26 usually catalyzed the polyubiquitin of its functional targets, such as ZEB1 and SOX2.^84,85^ Our findings established a novel link between TRIM26 and LKB1 upon the engagement of RBM4, implying that presumably TRIM26 may be involved in the related metabolic processes or metabolic adaptations.

Glutamine uptake and metabolism are required by cancer cells for sustaining oncogenic growth.^8,65,67^ Elevated Glutamine metabolism was found in ESCC samples as compared to the normal esophageal tissues.^5,10^ Deregulated Glutamine metabolism genes are correlated with a short survival for patients with esophageal cancers (5). Recent findings have supported that LKB1 deficiency rendered cells malignant activity and addicted to glutamine for survival maintenance in certain genetic backgrounds.^22,24,25^ While analyzing TCGA ESCC dataset, we found that few mutation sites or copy number loss occurred in *STK11* gene, which encodes LKB1, in ESCC. Yet, our results showed LKB1 protein level is downregulated in a large proportion of ESCC patients and negatively correlated with RBM4 expression. We found the RBM4 depletion-restricted glutamine utilization was mostly overcome by LKB1 reduction, which was previously reported to accelerate glutamine uptake and glutaminolysis.^25^ Pharmacological inhibition of AMPK, a critical LKB1 downstream kinase, has the similar restorative effect to LKB1 reduction, suggesting LKB1-AMPK axis may serve as a mediator in glutamine dependency of RBM4 high ESCC. This is also consistent with several studies that have linked AMPK inactivation to glutamine addiction.^66,86–88^ Nonetheless, the detailed mechanistic basis of glutamine addiction rendered by LKB1 or AMPK depletion is still less understood. Other than relying on LKB1-AMPK pathway, whether RBM4 directly modulates the expression of key genes in glutaminolysis remains to be explored.

In the pre-neoplastic phase, cellular senescence was able to respond diverse stressors to prevent tumorigenesis.^33,34,71^ Herein, our finding uncovered that RBM4 could be upregulated in pre-cancerous cells or cancer cells to evade senescence and maintain malignancy via suppressing LKB1 activity. Additionally, RBM4 knockdown contributes to cellular senescence via activating LKB1-AMPK-P27 pathway, and exogenous glutamate replenishment significantly reduces the population of senescent cells. This implies that, other than the cyclin-dependent kinase inhibitor, limited glutamine usage might be also responsible for RBM4 knockdown-induced pre-mature senescence, which is likely in part due to imbalance of redox homeostasis and occurrence of oxidative damage in the NADPH- and GSH-deficient environment. However, the relationships between glutamine metabolism and cellular senescence seem to be highly context and warrant of further investigation. For instance, BRAF V600E-induced senescence was accompanied by decreased glutamine usage.^89^ In another cellular senescence model named n-Sen hHCA2, the expression of kidney‑type glutaminase, which is used for converting glutamine to glutamate, is enhanced to satisfy ammonia production.^68^ In this study, we suggested that the ESCC cells with high expression of RBM4 and its concomitant inhibition of LKB1 activity have a stronger ability to fuel glutamine, which may assist stressed cells to escape from pre-mature aging and maintain a high proliferative rate, providing a new perspective for evaluating the mutual relationship of glutamine metabolism and cellular senescence.

As the main material sources of biomass and energy synthesis, glutamine is essential for maintaining rapid tumor growth and metastasis and redox homeostasis.^65,90,91^ Glutamine-derived metabolites also participate in the cancer-promoting cellular cascades, such as chromatin reorganization and hypoxia response.^92–94^ The conversion of glutamine to glutamate is catalyzed by glutaminase (GLS), which is frequently upregulated in some types of cancer cells.^8,95,96^ The higher demand and dependency of glutamine make such cancer cells more vulnerable to glutamine deprivation as compared to normal cells.^7,97,98^ Based on this, the glutaminase inhibitor, CB-839 has been exploited and showed efficacy and safety in Phase I clinical trials. In addition, only certain cancers are sensitive to GLS inhibition, but current knowledge on biomarkers for selection of patients with tumors dependent on glutamine pathway is poor, which have hindered effective clinical application of GLS inhibitors.^99–101^ Herein, our study showed that ESCC cells were more sensitive to glutamine pathway inhibition relative to normal esophageal epithelial cells, and the ESCC cells with aberrant expression of RBM4 exhibit higher glutamine dependency, offering the therapeutic potential of targeting glutaminolysis. The glutaminase inhibitor CB-839 showed substantially better efficacy in the treatment of ESCC xenograft tumors harboring RBM4 high expression. These findings provide novel mechanistic insights into ESCC progression depending on glutamine metabolism and uncover RBM4 as a potential biomarker and causative factor for targeting glutamine addiction in the diagnosis and treatment of ESCC.

## Materials and methods

### Ethics approval statements

All animal research was approved by the Institutional Animal Care and Use Committee of Dalian Medical University and the ethics approval number for animal work was AEE21015. The ethics approval statements for human samples were provided by the Ethics Committee of the Fourth Hospital of Hebei Medical University and the approval number was 2021KY080. Informed consents were obtained from all participants before the study.

### Cell culture and transfection

Esophageal squamous cell carcinoma (ESCC) cell lines YES2, KYSE30, KYSE70, KYSE140, KYSE150, KYSE180, KYSE410, KYSE450 and KYSE510 were kindly provided by Dr. Y. Shimada (Kyoto University). HEK293T cells were purchased from the American Type Culture Collection (ATCC). HEK293T cells was maintained in DMEM with 10 % FBS and ESCC cells were maintained in RPMI-1640 medium supplemented with 10% FBS. Cells were cultured in incubator with 5% CO2 at 37 °C. To stably overexpress or knockdown RBM4 in ESCC cells, lentiviral vectors were used. We used lipofectamine 3000 to transfect HEK293T cells with pCDH-HA-RBM4 (pCDH-HA empty vector as control) or pLKO.1- RBM4 (pLKO.1 empty vector as control) together with psPAX2 and pMD2.G. The supernatant media containing lentivirus were collected by centrifugation to remove any cellular contaminant after 48 h. Further, ESCC cells were infected with the viral particles, and the stably integrated cells were selected with 3 µg/ml puromycin for 5 days. Cells were maintained in medium containing 2 µg/ml puromycin. For CRISPR knockout cell construction, sgRNA was introduced into the lentiCRISPRv2 vector and packaged in lentivirus. To obtain the stably integrated cells, infected cells were screened with 3 µg/ml puromycin for 7 days. All the stable cell lines were validated by western blot assays before further analysis. The siRNAs and negative control (si-NC) were synthesized by RiboBio Co. (Guangzhou China). Lipofectamine 3000 (Thermo Fisher Scientific) was used to perform transfection according to the manufacturer’s instructions. The sequences of siRNA are listed in Supplemental Table1.

### STR profiling

NE2 and NE3 cell lines were kindly provided by Dr. Qimin Zhan, Shenzhen Bay Laboratory. These two epithelial cell lines were originally designated by the lab of Professor Sai-Wah Tsao (Department of Anatomy, University of Hong Kong, Hong Kong, China) and established from normal human esophageal tissue and immortalized. In addition, we have performed short tandem repeats (STRs) profiling to validate the two cell lines’ identity. According the identification reports, the cells named NE2 and NE3 are derived from human and have no cross-contamination of other human cell line. The DNA profiles of NE2 and NE3 are not included in ATCC and DSMZ databases, so the two STR profiles did not match any known cell line and could not be authenticated. However, while comparing the 8 core STR loci in our reports with a previous study depicting the characteristics of STRs in NE2 and NE3 provided by Professor Sai-Wah Tsao, the number of repeating units in alleles of D13S317, D7S820, D16S539, TPOX, TH01, CSF1PO, vWA and D5S818 in NE2/NE3 is completely consistent, suggesting NE2 and NE3 in our study have not changed or replaced. The NE2 cell line was authenticated using STR fingerprinting carried out by Beijing Microread Genetics Co., Ltd. The NE3 cell line was authenticated by STR fingerprinting by GENETIC TESTING BIOTECHNOLOGY.

### Clinical tissues samples collection

With the informed consent of primary ESCC patients who underwent tumor resection at the Fourth Hospital of Hebei Medical University, tumor and adjacent (more than 5 cm from the edge of the cancer focus) tissues were randomly collected, and a portion of the fresh tissues were stored in a -80 °C refrigerator, while the other portion were fixed in 4% paraformaldehyde and then embedded in paraffin. All patients had no history of preoperative radiotherapy and chemotherapy, and postoperative pathological diagnosis was positive. This study was approved by the Ethics Committee of the Fourth Hospital of Hebei Medical University (approval no. 2021KY080).

### Plasmid construction

To generate the mammalian expression plasmids pCDH-Flag-RBM4, pCDH-HA-RBM4, pCDH-Flag-P27, pCDH-Flag-MO25, pCDH-HA-STRAD, pCDH-Flag-LKB1, pCDH-HA-LKB1, pCDH-HA-TRIM26 and pCDH-Flag-TRIM26, the respective cDNAs were PCR amplified and then cloned into the lentivirus vector pCDH-CMV-MCS-EF1-Puro with Flag tag or HA tag with restriction enzymes NheI and NotI. The shRNAs targeting RBM4 were cloned into the pLKO.1 plasmid vector and shRNAs targeting P27, AMPK and LKB1 were cloned into the pGIPZ plasmid vector. All constructs were confirmed by DNA sequencing. Primers for PCR amplification, shRNAs and sgRNAs are listed in Supplemental Table 1.

### High throughput mRNA-sequence and data analysis

Total RNAs of RBM4 stably knockdown or control KYSE150 cells were isolated using Trizol™ method and subsequently cleaned using RNAeasy Kit (Qiagen). The DNA was removed from total RNAs by digesting in column with RNase-free DNase according to manufacturer’s instruction. Illumina TruSeq Total RNA Sample Prep kits were used to further purify polyadenylated RNA from total RNA. Ribosomal RNA was removed by Epicentre Ribo-zero™ rRNA removal Kit (Epicentre, USA). Three biological replicates were used for library construction and RNA-sequencing performed by Novogene Bioinformatics Technology. The DAVID online database was used for KEGG analysis.

### Western blot

Cells were harvested with RIPA lysis buffer containing 1 mM Na3VO4, 1 mM Cocktail and 1 mM PMSF. Cell debris was removed by centrifugation, then the protein samples were cooked at 95°C for 5 min in 1 × SDS sample buffer. An equal amount of protein samples was loaded into 10% SDS-PAGE for electrophoresis and then transferred to cellulose nitrate membrane. Enhanced chemiluminescence (Tanon) and MiniChemTM Chemiluminescence imager (SAGECREATION, Beijing) were used to detect the relative expression of proteins. The dilution ratio of primary antibodies for western blot was 1:1000. p-Rb(ser795,#9301), cyclinD3(DCS22), cyclinD1(E3P5S), CDK4(D9G3E), CDK6(DCS83), P53(7F5), P21(12D1), P27(D69C12), p-4EBP1(Thr37/46, 236B4), p-S6K(Thr389, 108D2), p-AMPKα(Thr172, D4D6D), AMPKα(DCS22), LKB1(27D10), LKB1(D60C5F10), PARP(46D11) antibodies were obtained from Cell Signaling Technology (Shanghai, China). Vinculin (66305-1-Ig), RBM4(11614-1-AP), HA(51064-2-AP) and GAPDH(60004-1-Ig) antibodies were obtained from Proteintech Group (Proteintech, China). MO25(ab51132) and STRAD (ab192879) antibodies were obtained from Abcam (Abcam, Shanghai, China). The anti-Flag (F3165) antibody was obtained from Sigma-Aldrich (Sigma-Aldrich, Shanghai, China).

### RNA extraction, cDNA synthesis and real-time PCR

Cells were lysed by Trizol (RNAiso Plus, Takara, Cat#9109), and extracted according to standard protocols. RNA samples were reverse-transcribed to cDNA using reverse transcriptase (YEASEN Biotech Co., 11141ES10), and RT-PCR was performed using the MonAMPTM ChemoHS Specificity Plus qPCR Mix (Low ROX, MQ00601) and the QuantStudio3 Real-Time PCR Instrument (Thermo Fisher Scientific). Primers for qRT-PCR are listed in Supplemental Table 2.

### Xenograft assays

The Institutional Animal Care and Use Committee of Dalian Medical University approved the experimental protocols performed on the animals. Nude mice were purchased from Beijing Vital River Laboratory Animal Technology. For tumor xenograft models, mice were subcutaneously implanted with 1.5 × 10^^6^ KYSE30 or KYSE150 with knockdown of RBM4 or empty vector. Ten mice were assigned to each group. Tumor size was measured by caliper every 2 or 3 days until mice were sacrificed and xenografts were removed and weighed and subjected to further analysis.

### Cell viability assay

Cell viability of KYSE150, KYSE30 and KYSE450 cells with stable expression or knockdown of RBM was measured by CCK-8 assays (APExBIO, K1018, American). Cells were seeded in 96-well plates at a density of 1.5–2 × 10^^6^ cells per well. After treatments, the culture medium was discarded. Then, 10 µL of CCK-8 reagent and 100 µL of medium were added to every well. Cells were cultured for 2 h in incubator with 5% CO2 at 37 °C, and the absorbance at 450 nm was detected with a spectrophotometer (TECAN, Switzerland). For rescue assays, cells were co-infected with the indicated viral particles for 24 h, and then the stably integrated cells were selected with 3 µg/ml puromycin for 5 days. After screening, the cells were maintained in medium containing 2 µg/ml puromycin and subjected to CCK8 assays to measure cell growth. For drug treatment, an equal number of RBM4-delpleted KYSE150 cells was seeded in 96-well plates. The growth curve of cells incubated with or without glutamic acid (0.5 mM), msethyl pyruvate (10 mM) or NAC (3 mM) was evaluated over 5 days in the CCK8 assay.

### EdU assay

KYSE150, KYSE30 and KYSE450 cells with overexpression or depletion of RBM4 were seeded in 96-well plates for 24 h and then treated with 50 μM EdU reagent for 2 h. Next, cells were treated according to the manufacturer’s protocol of Cell-Light EdU Apollo488 In Vitro Kit (RiboBio). Cells were visualized and imaged by Leica microscope (Leica Mi8, Germany)

### Colony formation assay

The stable RBM4 knockdown ESCC cells were seeded in 60 mm dishes (1 × 10^^3^ cells per dish) and incubated at 37 °C, 5% CO2 in humidified incubator for 2 weeks (updated with fresh medium every 3 days). Each treatment was carried out in triplicate. Colonies were fixed with 4% paraformaldehyde and stained with crystal violet solution. For rescue assays, cells were co-infected with viral particles expressing RBM4 shRNA and LKB1/AMPK/P27 shRNA for 24 h, and then the stably integrated cells were selected with 3 µg/ml puromycin for 5 days. After screening, the cells were maintained in medium containing 2 µg/ml puromycin and used for colony formation assays.

### Cell migration and wound-healing assays

Migration assay was performed in a 24-well plate with 8.0 µm polycarbonate membrane inserts (Corning). In brief, 1 × 10^^5^ KYSE 150 and KYSE 30 cells with overexpression of RBM4 were seeded into the upper chambers supplemented with serum-free medium and the lower chambers were filled with 20% FBS medium as a chemo-attractant. Cells on the lower surface were fixed, stained and counted during 24–48 h after seeding. Wound-healing assays were also performed to examine cell migration ability. 8 × 10^^5^ KYSE150, 8 × 10^^5^ KYSE450 and 6 × 10^^5^ KYSE 30 cells were seeded into 6-well plate and then scratched with medium size pipette tips. Then, cells were maintained in medium with 2% FBS. Scratched wound was monitored and pictures were taken at the indicated time points. The distances between the two edges of the scratched wound were measured using Image J software.

### Senescence-associatedβ-galactosidase (SA-β-Gal) staining

The activity of SA-β-Gal was determined using SA-β-Gal Staining Kit (Beyotime, C0602, Shanghai, China) according to manufacturer’s instructions. The cells were seeded in 6-well plates for 24–48 h and then washed twice with PBS followed by fixation with 4% paraformaldehyde at room temperature for 15 min. Next, the cells were washed twice with PBS, and incubated with freshly prepared staining solution at 37 °C without CO2 for 12–16 h. The SA-β-Gal-positive blue cells were observed under a light microscope (Nikon inverted microscope, Japan), and quantified by Image J program. For rescue assays, cells were co-infected with the indicated lentiviral particles for 24 h, and then the stable cells were selected with 3 µg/ml puromycin for 5 days. After screening, the cells were maintained in medium with 2 µg/ml puromycin and subjected to SA-β-gal staining. For drug treatment. the KYSE150 cells with stable knockdown of RBM4 were cultured in medium supplemented with glutamic acid (0.5 mM), methyl pyruvate (10 mM), NAC (3 mM) or solvent control. After 3 days, cells were seeded in 6-well plates for SA-β-gal activity assay.

### Immunoprecipitation assay

For immunoprecipitation assays, KYSE150 cells were co-transfected plasmids according to the requirements of the experiment in the article for 24–48 h using LipoPlus reagent (Sage) according to the manufacturer’s instructions. Cells in the presence or absence of Bortezomib (PS341; Selleck; Cat#S1013) were lysed by IP lysis buffer (50 mM Tris-HCl pH 7.4, 150 mM NaCl, 1 mM EDTA, 1% TritonX-100, PMSF and Cocktail). Cell lysates were incubated with anti-Flag M2 Affinity Gel (Sigma Aldrich) at 4 °C overnight. Following washes with 1 ml of washing buffer (50 mM Tris-HCl pH 7.5, 150 mM NaCl, 0.5% TritonX-100, 10% Glycerol), eluted protein samples was subjected to western blotting.

### Immunofluorescence assay

Cells were seeded in 6-well plates on coverslips for 24 h. Cells were fixed in 4% paraformaldehyde for 15 min, followed by cell permeabilization with 0.2% TritonTMX-100 for 10 min. The cells were washed with PBS three times/ 5 min. Cells were blocked by 3% BSA for 10 min and incubated overnight at 4 °C in primary antibodies (anti-Flag tag, F3165, 1:10000, sigma) (anti-HA tag, H6908, 1:5000, sigma) diluted in 3% BSA. Secondary antibodies labeled with Alexa fluorophore 488/592 were diluted 1:500 in 3% BSA and incubated for 1 h at room temperature. Nuclear was staining with DAPI. Images were captured with a Leica laser confocal microscope.

### Glutamine uptake assay

The glutamine consumption levels of NE2, A549, ESCC cells and stable RBM4-overexpressing or knockdown KYSE150, KYSE30 and KYSE450 cells were determined by comparing glutamine levels in medium alone to the cell culture medium using a commercially available colorimetric determination kit (Glutamine Detection Assay Kit, ab197011, Abcam, Cambridge, UK) following the manufacturer’s instructions. The assay is based on the hydrolysis of glutamine to glutamate producing a stable signal which is directly proportional to the amount of glutamine in the sample. Experimental values were interpolated in Excel within a standard curve of 0, 2, 4, 6, 8, and 10 nmol/well. For rescue experiments, RBM4 stable knockdown cells were treated with AMPK inhibitor (5 µM) or DMSO (vehicle) at 24 h after seeding an equal number in 10 cm dishes. After 36 h, the decrease of glutamine was examined.

### Glutamate production assay

The relative levels of glutamate in NE2, A549, stable RBM4-overexpressing or knockdown ESCC cells treated with or without deprivation of glutamine were detected using a commercially available colorimetric determination kit (Glutamate Detection Assay kit, ab83389, Abcam, Cambridge, UK) following the manufacturer’s instructions. For rescue experiments, an equal number of stable cells were seeded in the culture dish and culturing in the medium with DMSO or AMPK inhibitor (5 µM). After 36 h, the decrease of glutamine was examined. For rescue experiments, RBM4 stable knockdown cells were treated with AMPK inhibitor (5 µM) or DMSO (vehicle) at 24 h after seeding an equal number in 10 cm dishes. After 36 h, the levels of intracellular glutamate were examined.

### GSSG/GSH assay

GSSG and GSH levels were measured using a GSSG/GSH Quantification Kit II (Dojindo, G263). Briefly, the medium was removed and 2 × 10^^7^ cells were harvested. 40 µl GSSG standard solution, GSH standard solution or sample solution was added to 96-well plate, then 60 µl buffer solution was added to each well and incubated at 37 °C. After 1 h, 60 µl Substrate Working solution and Enzyme/Coenzyme Working Solution and incubated for 10 min at 37 °C. The absorbance was measured at 405 nm by a multifunctional plate reader (Enspire 2300, PerkinElmer).

### IC50 assay

To identify the half-maximal inhibitory concentration (IC50) of NE2, NE3 and ESCC cells and stable RBM4-overexpressing A549 or ESCC cells compared to control group against glutaminase inhibitor CB-839, the cells were seeded with equal amount into 96-well plates in triplicates, and serial dilutions of CB-839 (Selleck; Cat#S7655) were added 24 h later. After 72 h of drug treatment, cell viability was measured by CCK-8 assays. A dose-response model was used to estimate half-maximal inhibitory concentration values from cell viability data.

### Immunohistochemistry

ESCC tissue arrays purchasing from Outdo Biotech (Shanghai, China) and xenografts’ tumor tissues were used for immunohistochemistry (IHC) staining. Tumor tissues from in vivo therapy experiments on KYSE30 tumor-bearing nude mice with CB-839 were fixed with 4% formaldehyde and then embedded in paraffin blocks before being processed for immunohistochemical analysis. Tissues were subject to heat-induced antigen retrieval using 10 mM sodium citrate buffer followed by 3% H2O2 for 30 min to quench endogenous peroxidase activity. Sections were incubated overnight at 4 °C with following antibodies (RBM4, 1:200; LKB1, D60C5F10, 1:50; P27,1:200; Ki67, 1:200; p-AMPK, 1:200) and detected using Vectastain Elite ABC kit and DAB Peroxidase Substrate kit as per the manufacturer’s protocol. The H-score was a histological scoring method for processing immunohistochemistry that converted the number of positively stained tumor cells and their staining intensity into corresponding values for the purpose of quantification of tissue staining. H-SCORE = ∑(pi × i) = (percentage of weak intensity × 1) + (percentage of moderate intensity × 2) + (percentage of strong intensity × 3), where pi indicated the percentage of positive signal pixel area/number of positive tumor cells; i represents the coloring intensity. The positively stained cells were graded as 0 (no positive tumor cells), 1 (<10%), 2 (10–50%), and 3 (>50%). The staining intensity was determined as 0 (no staining); 1 (weak staining = light yellow), 2 (moderate staining = yellow brown), and 3 (strong staining = brown). As for IHC analysis on ESCC tissue array, the results were analyzed by the pathologist who provided a value ranging from 0 to 3 for each sample. “0” indicates negative staining; “1” means weak positive staining; “2” denotes moderately positive staining, and “3” indicates strong positive staining. The groups with negative and weak positive staining were considered as low expressing groups, and groups with moderately positive and strong positive staining were considered as high expressing groups. For analysis of mass spectrometry-based proteomic profiling of ESCC tumors and adjacent non-tumor tissues (PXD021701), Cutoff Finder was used to determine a cutoff point and stratify patients into two groups (RBM4 high/ LKB1 low and RBM4 low/LKB1 high expression) based on the relative protein expression (RBM4: 0.7054, LKB1: 0.05854).

### In vivo therapy on tumor-bearing nude mice with CB-839

The Institutional Animal Care and Use Committee of Dalian Medical University approved the experimental protocols performed on the animals. Four- to six-week-old male BALB/c-nu (WT) mice were purchased from Beijing Vital River Laboratory Animal Technology Co., Ltd, China. A total of 5 × 10^^6^ viable RBM4 stable overexpression or control KYSE30 cells were subcutaneous injected into the underarm and growth was monitored every 3 days. When the tumor volume grew into 50–250 mm^3^, RBM4 overexpression and control mice were randomly divided into four groups respectively. Mice were treated with CB-839 200 mpk, by oral gavage, every 12 h. Tumor volumes were measured every 3 days. After 21 days of treatment, the mice were sacrificed to remove and weigh the xenografted tumors, which were used for western blotting and IHC analysis.

### TCGA and GEO database analysis

To validate the distinct expression of RBM4 in ESCC, we have analyzed the data from TCGA database containing 162 tumor and 11 normal unmatched esophageal samples. Differential expression of RBM4 between ESCC and normal samples from esophageal columnar epithelium was assessed by the analysis of two ESCC microarray datasets from the NCBI GEO database. GSE161533 included 28 tumor tissues and 56 normal tissues, while GSE26886 included nine tumor tissues and 19 normal tissues. The significance was determined by unpaired Student’s t-test.

### Statistics

Data are presented as mean ± SD or mean ± SEM as required. Statistical significance was determined by two-tailed Student’s *t*-test, one-way ANOVA with Dunnett multiple comparisons, two-way repeated measures ANOVA according to different situation. Two-tailed Student’s *t*-test was applied to determine statistical significance for two-group comparison. When comparing multiple groups to the same control, one-way ANOVA with Dunnett multiple comparisons was used. In cellular, tumor growth curve experiments or survival curves of patients with high/ low expression of RBM4, two-way repeated measures ANOVA was applied. *P < 0.05, **P < 0.01, ***P < 0.001, ****P < 0.0001.

## Supplementary information


Supplementary_Materials


## Data Availability

The authors declare that all the data supporting the findings of this study are available within the article and its Supplemental information files. RNA-seq data in this study have been deposited in Gene Expression Omnibus of NCBI with the accession code GSE220613.
