# Correction: RBM4 dictates ESCC cell fate switch from cellular senescence to glutamine-addiction survival through inhibiting LKB1-AMPK-axis

**DOI:** 10.1038/s41392-026-02975-z

**Published:** 2026-08-31

**Authors:** Lei Chen, Wenjing Zhang, Dan Chen, Quan Yang, Siwen Sun, Zhenwei Dai, Zhengzheng Li, Xuemei Liang, Chaoqun Chen, Yuexia Jiao, Lili Zhi, Lianmei Zhao, Jinrui Zhang, Xuefeng Liu, Jinyao Zhao, Man Li, Yang Wang, Yangfan Qi

**Affiliations:** 1 Institute of Cancer Stem Cells and the Second Affiliated Hospital of Dalian Medical University, Dalian Medical University, Dalian, 116044 China; 2 Department of Pathology, the First Affiliated Hospital of Dalian Medical University, Dalian, 116011 China; 3 Department of Oncology, the Second Affiliated Hospital of Dalian Medical University, Dalian, 116023 China; 4 Department of Thoracic Surgery, the First Affiliated Hospital of Dalian Medical University, Dalian, 116011 China; 5 Research Center, the Fourth Hospital of Hebei Medical University, Shijiazhuang, 050011 China

Correction to: *Sig Transduct Target The* https://doi.org/10.1038/s41392-023-01367-x, published online 21 April 2023

After online publication of the article,^1^ the authors identified an inadvertent error in Figure 6j. During the assembly of the figure, a single representative image was mistakenly duplicated to show both the RBM4 sh1 + NAC and RBM4 sh1 + glutamic acid conditions. The correct image for the RBM4 sh1 + NAC condition has now been inserted. This correction does not alter the study’s overall conclusions. The amended Figure. 6j is provided below.

**Figure:**
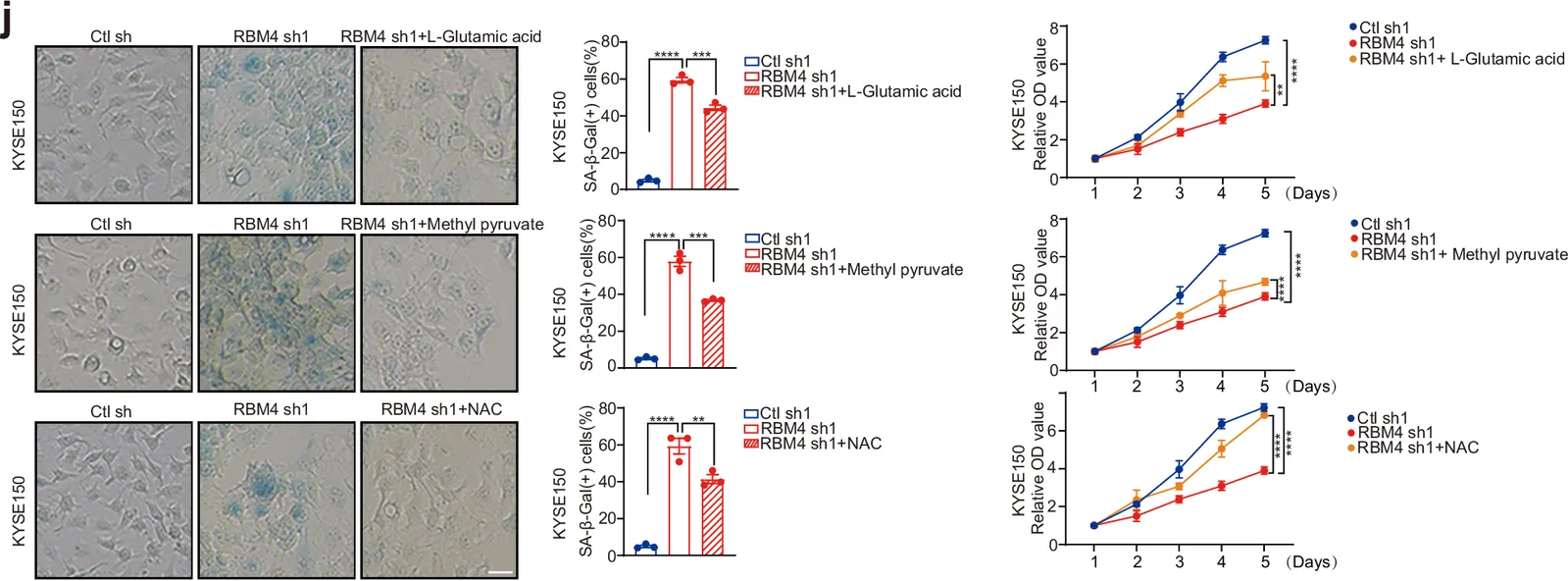

**Fig. 6j**. SA-β-gal staining of KYSE150 cells with stable knockdown of RBM4 in the presence or absence of glutamic acid (0.5 mM), methyl pyruvate (10 mM), or NAC (3 mM). Three experiments were performed, and the mean ± SD percentage of SA-β-gal-positive cells is shown. *P* values were determined by one-way ANOVA followed by Dunnett’s multiple-comparisons test. Scale bar, 25 μm.
