# Supplementary material for: RBM4 dictates ESCC cell fate switch from cellular senescence to glutamine-addiction survival through inhibiting LKB1-AMPK-axis
Source: Signal Transduct Target Ther. 2023 Apr 21;8:159. doi: 10.1038/s41392-023-01367-x (PMC10119322; doi:10.1038/s41392-023-01367-x)
Supplement: Supplementary file 1 — Supplementary_Materials [file 41392_2023_1367_MOESM1_ESM.docx]

Supplementary Materials for

RBM4 dictates ESCC cell fate switch from cellular senescence to glutamine-addiction survival through inhibiting LKB1-AMPK-axis

Lei Chen^1#^, Wenjing Zhang^1#^, Dan Chen^2#^, Quan Yang^1#^, Siwen Sun^3#^, Zhenwei Dai^1^, Zhengzheng Li^1^, Xuemei Liang^4^, Chaoqun Chen^1^, Yuexia Jiao^1^, Lili Zhi^1^, Lianmei Zhao^5^, Jinrui Zhang^1^, Xuefeng Liu^1^, Jinyao Zhao^1^, Man Li^3*^, Yang Wang^1*^, Yangfan Qi^1*^

Correspondence to: [yangfanqi@dmu.edu.cn](mailto:yangfanqi@dmu.edu.cn); [yangwang@dmu.edu.cn](mailto:yangwang@dmu.edu.cn);

liman126126@163.com

**This PDF file includes:**

Supplementary Figure 1-6 with their legends

Supplementary Table 1-2

All original films of Western blots

**
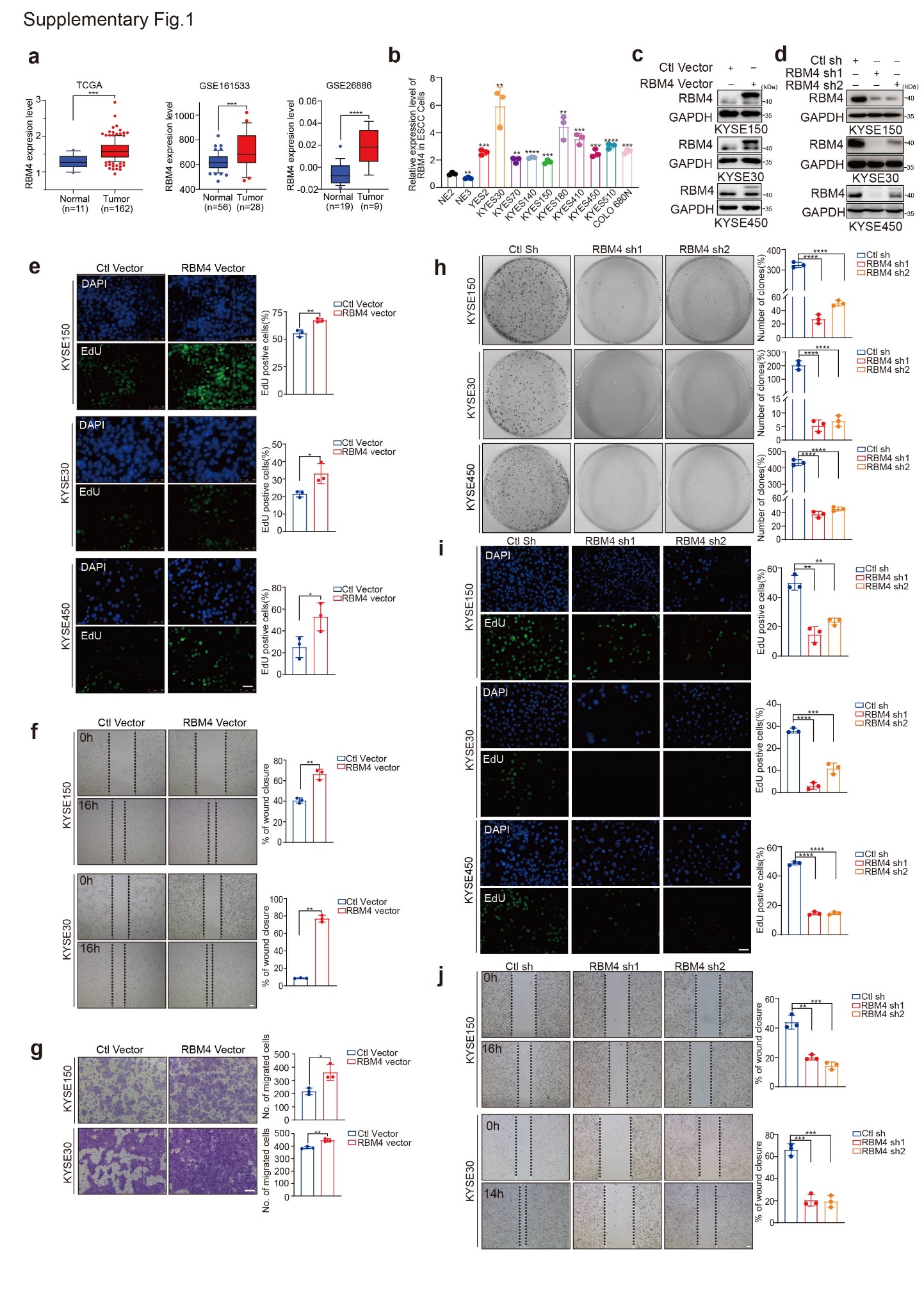
 Supplementary Figure 1**

**Supplementary Fig. 1** **RBM4 exhibits tumor-promoting activity in ESCC cells. (a)** Left panel: the differential expression of RBM4 was analyzed in ESCC (n =162) and normal unmatched esophageal samples (n = 11) from The Cancer Genome Atlas (TCGA) database. Middle and right panels: differential expression of RBM4 between ESCC and normal esophageal squamous epithelium was assessed by the analysis of two ESCC microarray datasets from the NCBI GEO database (GSE161533: 28 tumor, 56 normal) (GSE26886: 9 tumor, 19 normal). Significance was determined by unpaired Student’s t test. *** *P* < 0.001, **** *P* < 0.0001. **(b)** The mRNA expression level of RBM4 in normal esophageal epithelial cells and different ESCC cells was examined with the qRT-PCR approach. *P* values were determined by One-way ANOVA with Dunnett multiple comparisons. **(c-d)** The protein levels of RBM4 were determined in KYSE150, KYSE30 and KYSE450 cells with stable overexpression of RBM4 (c) or depletion of RBM4 (d) using western blotting. **(e)** The proliferation of different RBM4-overexpressed ESCC cells was examined by the EdU staining assay. Representative images and quantification of EdU positive cells were shown. Scale bar = 50 μm. Three experiments were performed and mean ± SD was plotted with *P* values determined by One-way ANOVA with Dunnett multiple comparisons. **(f)** The migration ability of different ESCC cells with RBM4-overexpression was examined by the wound-healing assay. Representative images and quantification of wound healing of the indicated cells were shown. Scale bar = 100 μm. Three experiments were performed and mean ± SD was plotted with *P* values were determined by One-way ANOVA with Dunnett multiple comparisons. **(g)** The migration ability of different ESCC cells with RBM4-overexpression was examined by the transwell assay. Representative images and quantification of transwell assays of the indicated cells were shown. Scale bar = 100 μm. Three experiments were performed and mean ± SD was plotted (*P* values were determined by One-way ANOVA with Dunnett multiple comparisons). **(h-i)** The proliferation of distinct RBM4-depleted ESCC cells was examined by the colony formation assay (h) and EdU staining assay (i). Representative images and quantification of EdU positive cells and colony formation assay were shown. Scale bar = 50 μm. Three experiments were carried out with mean ± SD of β-gal positive cells plotted (*P* values were determined by One-way ANOVA with Dunnett multiple comparisons). **(j)** The migration ability of different ESCC cells with RBM4-knockdown was examined by the wound-healing assay. Representative images and quantification of wound healing assays of the indicated cells were shown. Scale bar = 100 μm. Three experiments were carried out with mean ± SD of β-gal positive cells plotted. *P* values were determined by One-way ANOVA with Dunnett multiple ­­­­comparisons. * *P* < 0.05, ** *P* < 0.01, *** *P* < 0.001, **** *P* < 0.0001.

**
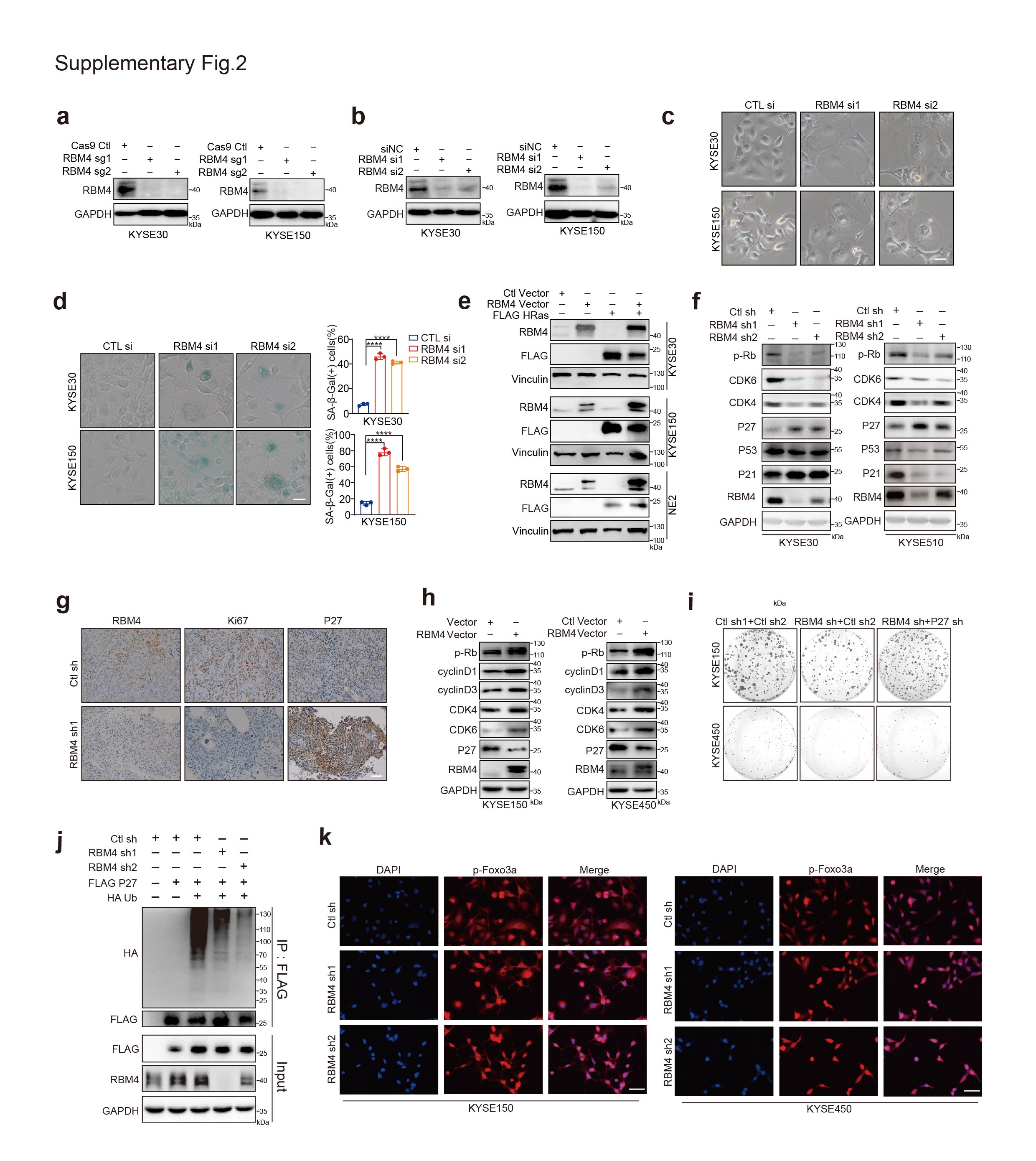
­­ Supplementary Figure 2**

**Supplementary Fig. 2** **Reduction of RBM4 induces_­­_ ESCC cellular senescence depending on P27 accumulation. (a-b)** The efficiency of RBM4 protein knockout/silencing by Cas9/sgRNA (a) or siRNA (b) was determined by western blot analysis. **(c)** The morphology changes of KYSE30 and KYSE150 cells transfected with ­­­siRNA targeting RBM4 or no-targeting control siRNA. Scale bar =25 μm. **(d)** β-gal staining of KYSE30 and KYSE150 cells transfected with siRNA targeting RBM4 or no-targeting control siRNA. Scale bar =25 μm. Three experiments were carried out with mean ± SD of β-gal positive cells plotted with *P* value determined by One-way ANOVA with Dunnett multiple comparisons. * *P* < 0.05, ** *P* < 0.01, *** *P* < 0.001, **** *P* < 0.0001. **(e)** Evaluation of overexpression efficiency of RBM4 and Flag-HRas by western blot assays. **(f)** The protein levels of RBM4, p-Rb, CDK6, CDK4, P53, P21, and P27 in RBM4-depleted KYSE510 and KYSE30 cells were examined in western blot assays. **(g)** The xenografted tumors generated from RBM4-depleted ESCC cells were applied to IHC staining with anti-RBM4, Ki67, and P27 antibodies. Scale bar = 100 μm. **(h)** The protein levels of RBM4, p-Rb, cyclin D1, cyclin D3, CDK6, CDK4, and P27 in ESCC cells with RBM4-overexpression were examined in a western blot assay. **(i)** The proliferation of distinct RBM4-depleted ESCC cells with or without P27 was examined by the colony formation assay. Representative images of colony formation assay were shown. **(j)** Immunoprecipitation assay was applied to examine the ubiquitination of P27 upon RBM4 depletion in the presence of PS341 (10 μM). The protein complexes were precipitated by anti-Flag antibody and analyzed through western blotting. **(k)** Immunofluorescence assay was utilized to determine the localization of p-Foxo3a in RBM4-depleted KYSE150 and KYSE450 cells. Scale bar = 50 μm.


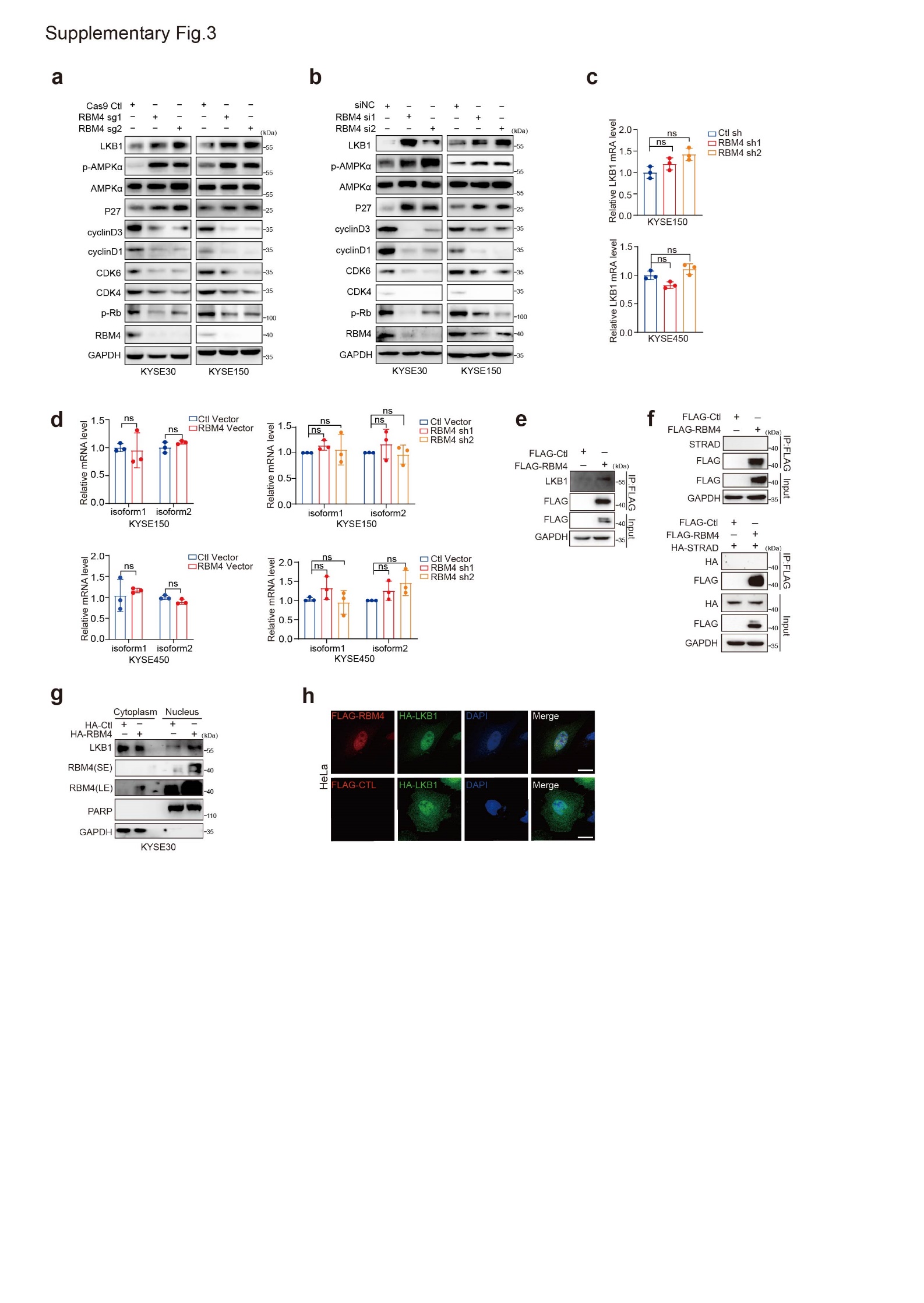
 **Supplementary Figure 3**

**Supplementary Fig. 3 RBM4 interacts with LKB1 in nucleus. (a-b)** The protein levels of LKB1, p-AMPKα, AMPKα, P27, cyclin D3, cyclin D1, CDK6, CDK4, p-Rb and RBM4 in KYSE30 and KYSE150 cells with RBM4 knockout/silencing by Cas9/sgRNA (a) or siRNA (b) were examined in western blot assays. **(c)** The mRNA levels of LKB1 in KYSE150 and KYSE450 cells with stable overexpression of RBM4 (left) or depletion of RBM4 (right) were examined using qRT-PCR approach. *P* values were determined by One-way ANOVA with Dunnett multiple comparisons, ns indicate not statistically significant. **(d)** The mRNA levels of LKB1 canonical isoform (isoform1) or LKB1 short isoform (isoform2) in KYSE150 or KYSE450 cells with stable RBM4 overexpression or knockdown or control vector were examined by qRT-PCR approach. *P* values were determined by One-way ANOVA with Dunnett multiple comparisons, ns indicate not statistically significant. **(e)** Immunoprecipitation assay was carried out to determine the interaction between Flag-RBM4 and endogenous LKB1. The protein complexes were precipitated by anti-Flag antibody and analyzed through western blotting. **(f)** Immunoprecipitation assay was carried out to determine the interaction between Flag-RBM4 and endogenous STRAD (upper) and HA-STRAD (lower). Flag-tagged precipitated complexes and lysates were analyzed by western blotting. **(g)** The levels of LKB1 and RBM4 were examined in the cytoplasm and nucleus of KYSE30 cells expressing HA-RBM4 by a nucleoplasmic fractionation assay. SE, short exposure; LE, long exposure. **(h)** Confocal immunofluorescence microscopy was utilized to examine the localization of Flag-RBM4 and HA-LKB1 in HeLa cells. Scale bar = 10 μm.

**
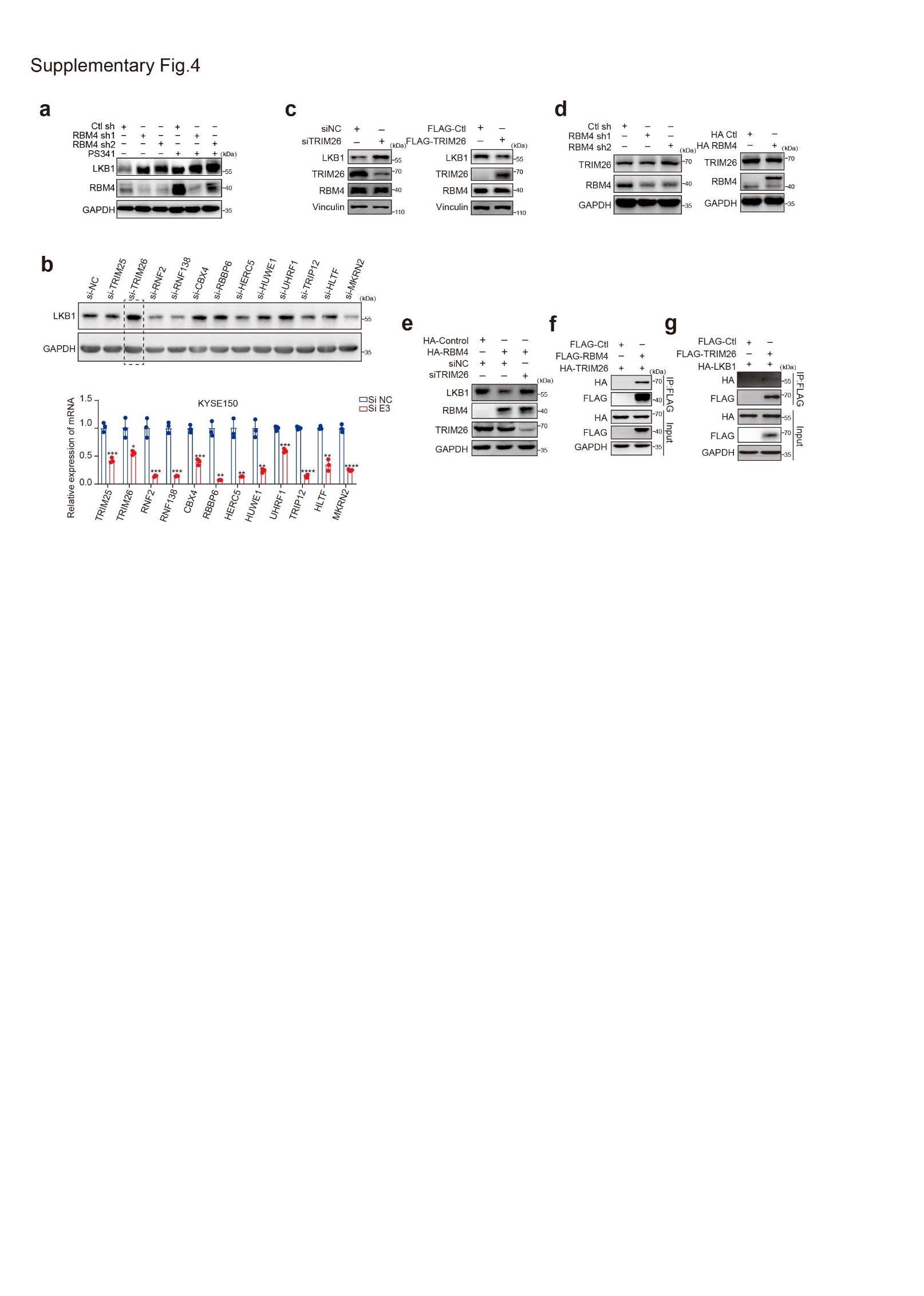
 Supplementary Figure 4**

**Supplementary Fig. 4 TRIM26 directly promotes RBM4-mediated LKB1 degradation. (a)** The protein levels of LKB1 in RBM4-depleted KYSE150 cells or control cells with or without PS341 treatment were examined using western blotting. **(b)** A western blot assay was applied to examine the level of LKB1 upon depletion of distinct RBM4-interacted E3 ligases (upper). The knockdown efficiency of different E3 ligases was identified by qRT-PCR approach (lower). Three experiments were carried out with *P* value determined by Student’s t test. * *P* < 0.05, ** *P* < 0.01, *** *P* < 0.001, **** *P* < 0.0001. **(c)** Protein levels of LKB1 and RBM4 were measured in KYSE150 cells upon TRIM26 knockdown or overexpression. **(d)** Protein levels of TRIM26 were measured in KYSE150 with stable depletion of RBM4 (left) or overexpression of RBM4 (right). **(e)** Protein levels of LKB1 were measured in KYSE150 cells overexpressing RBM4 in the presence or absence of TRIM26 knockdown. **(f)** Immunoprecipitation assay was carried out in KYSE150 cells expressing Flag-RBM4 and HA-TRIM26. The protein complexes were precipitated with anti-Flag followed by western blotting analysis. **(g)** Immunoprecipitation assay was performed in KYSE150 cells expressing Flag-TRIM26 and HA-LKB1, and the Flag-tagged precipitated-complexes were analyzed through western blotting.

**Supplementary Figure 5**

**
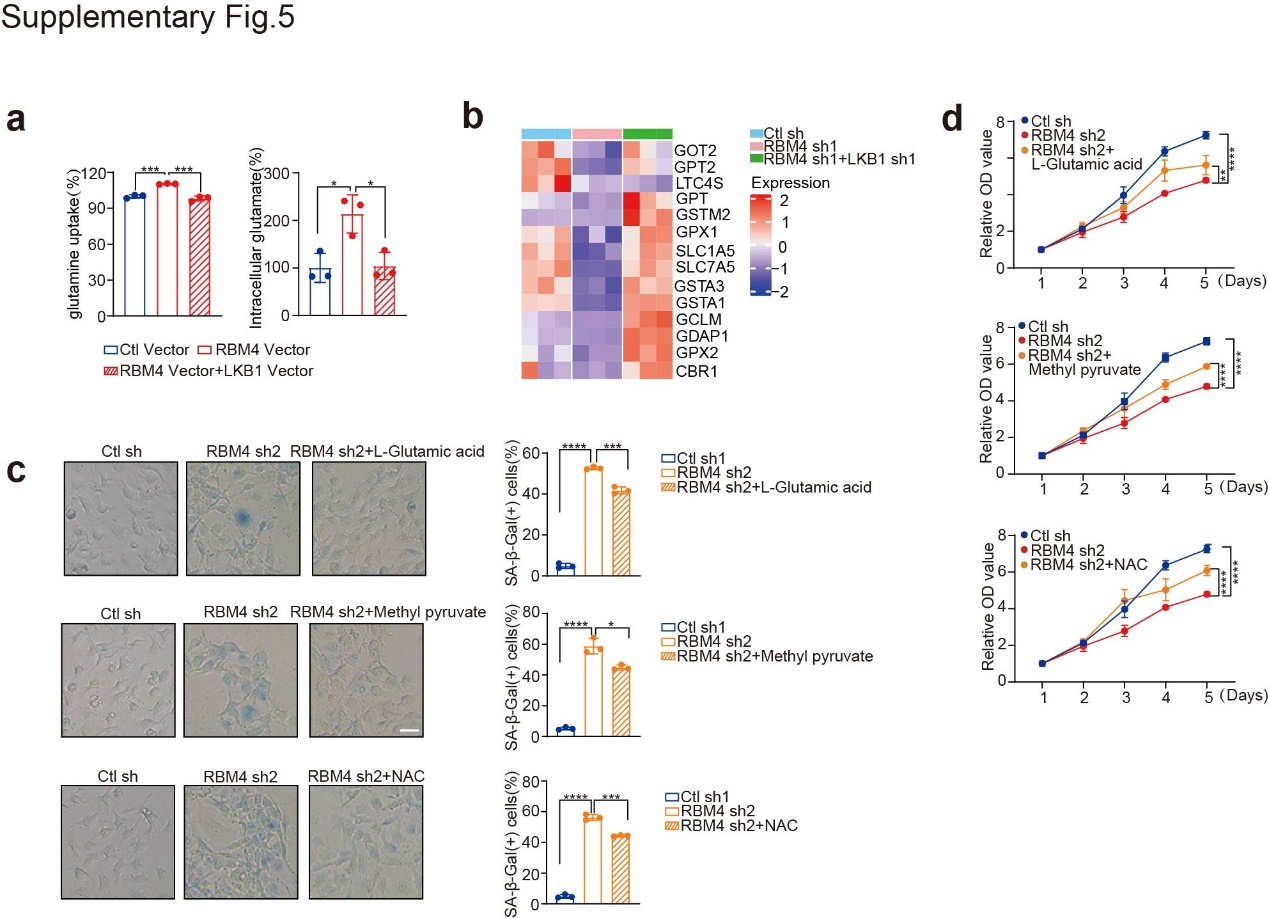
**

**Supplementary Fig. 5 Glutamine metabolism was enhanced by RBM4-LKB1 axis to sustain ESCC cells survival. (a)** The glutamine uptake and intracellular glutamate levels were examined in KYSE150 cell with stable co-transfection of RBM4 and LKB1. Three experiments were performed and mean ± SD was plotted. *P* values were determined by One-way ANOVA with Dunnett multiple comparisons. * *P* < 0.05, ** *P* < 0.01, *** *P* < 0.001. **(b)** Heatmap showing differences in RNA levels of metabolism-related genes between control vector and RBM4-depleted ESCC cells with LKB1 knockdown, quantified by qRT-PCR (*P* values were determined by One-way ANOVA with Dunnett multiple comparisons). **(c)** β-gal staining of KYSE150 cells with stable knockdown of RBM4 in the presence or absence of glutamic acid, methyl pyruvate or NAC. Three experiments were carried out with mean ± SD of β-gal positive cells plotted (*P* values were determined by One-way ANOVA with Dunnett multiple comparisons). Scale bar = 25 μm. **(d)** The growth curve of RBM4-depleted KYSE150 cells was measured by CCK8 assay in the presence or absence of glutamic acid, NAC, or methyl pyruvate. *P* values were determined by two-way repeated measures ANOVA. * *P* < 0.05, ** *P* < 0.01, *** *P* < 0.001, **** *P* < 0.001.


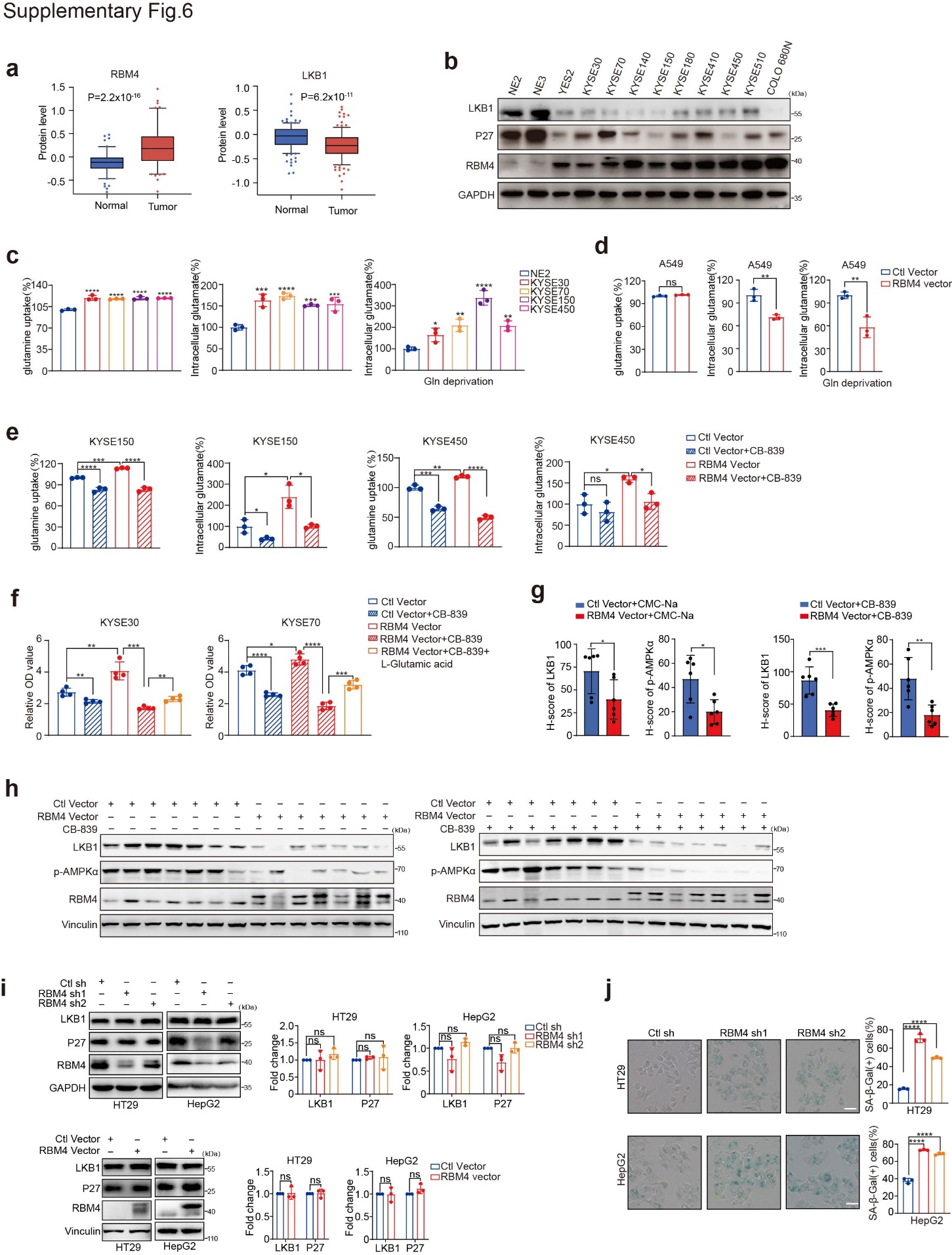
 **Supplementary Figure 6**

**Supplementary Fig. 6 RBM4 confers glutamine-dependency in ESCC through downregulating LKB1. (a)** The protein levels of RBM4 and LKB1 in ESCC and normal tissue were obtained from PXD021701. *P* values were determined by Student’s t test. **(b)** Protein levels of LKB1, RBM4 and P27 were examined in immortalized normal esophageal epithelial cells (NE2) and distinct ESCC cells. **(c)** The glutamine consumption and intracellular glutamate levels were measured in NE2, KYSE30, KYSE70, KYSE150 and KYSE450 cells. Three experiments were performed and mean ± SD was plotted (*P* values were determined by One-way ANOVA with Dunnett multiple comparisons). **(d)** The glutamine consumption and intracellular glutamate levels were examined in A549 cell with stable overexpression of RBM4.Three experiments were performed and mean ± SD was plotted (*P* values were determined by Student’s t test). **(e)** The glutamine consumption and intracellular glutamate levels were examined in KYSE150 and KYSE450 cells with stable overexpression of RBM4 in the presence or absence of the glutaminase inhibitor CB-839. Three experiments were performed and mean ± SD was plotted. *P* values were determined by One-way ANOVA with Dunnett multiple comparisons. **(f)** KYSE30 and KYSE70 cells with RBM4-overexpression or control vector were treated with CB-839 or solvent control (DMSO), and extra 0.5 mM L-glutamic acid was added the culture medium for the drug-treated cells. After 72 hours, the cell viability of each group was measured by CCK8 assay. *P* values were determined by One-way ANOVA with Dunnett multiple comparisons. **(g)** After 21 days of oral administration of CB-839 or vehicle (0.5% CMC-Na), the nude mice bearing RBM4-overexpressing or the empty vector xenografts were sacrificed to remove tumors for immunohistochemistry analysis. The semi-quantitative analysis of LKB1 and p-AMPK levels was performed by image J analysis from IHC staining. H scores are represented as mean ± SD, Student’s t test. H-Score = ∑(pi×i) = (percentage of weak intensity×1)+(percentage of moderate intensity×2)+(percentage of strong intensity×3), pi indicates the percentage of positive signal pixel area/number of positive tumor cells, i represents the coloring intensity. **(h)** 21-days after oral administration of CB-839 or vehicle (0.5% CMC-Na), the nude mice bearing RBM4-overexpressing or the empty vector xenografts were sacrificed to remove tumors for western blot analysis of RBM4, LKB1 and phosphorylation of AMPK. **(i)** Western blot analysis of protein levels of RBM4, LKB1, P27 in RBM4-depleted or overexpressing colorectal cancer and liver cancer cells. The gray values of LKB1 or P27 protein band normalized to that of the loading control were quantified and plotted as mean ± SD of three independent experiments, ns = non-significant. **(j)** β-gal staining of colorectal cancer and liver cancer cells with stable depletion of RBM4 or empty vector. Three experiments were carried out with mean ± SD of β-gal positive cells plotted. Scale bar = 25 μm. *P* values were determined by One-way ANOVA with Dunnett multiple comparisons. * *P* < 0.05, ** *P* < 0.01, *** *P* < 0.001, **** *P* < 0.0001.

**Supplementary Table 1**

| **siRNA sequences** | | |
| --- | --- | --- |
| **Name** | **Sense (5’-3’)** | **Anti-sense (5’-3’)** |
| siRBM4-1 | CCUUGAUAACACAGAGUUUTT | AAACUCUGUGUUAUCAAGGTT |
| siRBM4-2 | GCUGCCUCCGUGUAUAAUUTT | AAUUAUACACGGAGGCAGCTT |
| siTRIP12 | CCAGGAGCAACAACUGAAAUCUGCA | UGCAGAUUUCAGUUGUUGCUCCUGG |
| siHERC5 | CCACCACACCACAGAUUGUTT | ACAAUCUGUGGUGUGGUGGTT |
| siTRIM25 | GGCACAAACUAACUGUCAUTT | AUGACAGUUAGUUUGUGCCTT |
| siUHRF1 | AGACGGAAUUGGGGCUGUATT | UACAGCCCCAAUUCCGUCUTT |
| siRBBP6 | UCUGAUUCCUAAGAAUUCUUCUGUA | UACAGAAGAAUUCUUAGGAAUCAGA |
| siMKRN2 | UUGCCUUGCUCAAAGUAUUUACAGG | CCUGUAAAUACUUUGAGCAAGGCAA |
| siRNF2 | CGCCACUGUUGAUCACUUATT | UAAGUGAUCAACAGUGGCGUU |
| siHLTF | GGAAUAUAAUGUUAACGAUTT | AUCGUUAACAUUAUAUUCCTT |
| siCBX4 | GCAAGAGCGGCAAGUACUATT | UAGUACUUGCCGCUCUUGCTT |
| siRNF138 | CCUGUGUCAAGAAUCAAAUTT | AUUUGAUUCUUGACACAGGTT |
| siTRIM26 | CCGGAGAAUUCUCAGAUAATT | UUAUCUGAGAAUUCUCCGGTT |
| siHUWEI | UUGCUAUGUCUCUGGGACATT | UGUCCCAGAGACAUAGCAAUU |

**Supplementary Table 2**

| **Primers for mammalian expression plasmids** | |
| --- | --- |
| **Name** | **Primer sequence** |
| RBM4-NheI-F | AATGCTAGCATGGTGAAGCTGTTCATCGG |
| RBM4-NotI-R | ATTGCGGCCGCTTAAAAGGCTGAGTACCG |
| P27-NheI-F | AGTGCTAGCATGTCAAACGTGCGAGTG |
| P27-NotI-R | ATTGCGGCCGCTTACGTTTGACGTCTT |
| MO25-NheI-F | ACTGCTAGCATGCCGTTCCCGTTTGG |
| MO25-NotI-R | ATTGCGGCCGCTTAAGCTTCTTGCTG |
| LKB1-NheI-F | AATGCTAGCATGGAGGTGGTGGACCCG |
| LKB1-NotI-R | ATTGCGGCCGCTCACTGCTGCTTGCAG |
| STRAD-NheI-F | CCGGCTAGCATGTCATTTCTTGTAAGTAAACC |
| STRAD-NotI-R | ATTGCGGCCGCTCAGAACTCCCAATCG |
| TRIM26-NheI-F | AATGCTAGCATGGCCACGTCAGCCCCACT |
| TRIM26-NotI-R | ATTGCGGCCGCTCAGGGTCTTAGCAGGAG |

| **Primers for sgRNA** | |
| --- | --- |
| **Name** | **Primer sequence** |
| RBM4-gRNA-F1 | CACCGGCCCACATGCAACTTTGTTG |
| RBM4-gRNA-R1 | AAACCAACAAAGTTGCATGTGGGCC |
| RBM4-gRNA-F2 | CACCGTGTGTTATCAAGGCCCCTGA |
| RBM4-gRNA-R2 | AAACTCAGGGGCCTTGATAACACAC |

| **Primers for shRNA** | |
| --- | --- |
| **Name** | **Primer sequence** |
| RBM4 sh1-F | CCGGGCTGGAATGTGACATCATTAACTCGAGT  TAATGATGTCACATTCCAGCTTTTTTG |
| RBM4 sh1-R | AATTCAAAAAAGCTGGAATGTGACATCATTAA  CTCGAGTTAATGATGTCACATTCCAGC |
| RBM4 sh2-F | CCGGCCTGTTCTTCTGTCCTTCAATCTCGAGAT  TGAAGGACAGAAGAACAGGTTTTTTG |
| RBM4 sh2-R | AATTCAAAAAACCTGTTCTTCTGTCCTTCAATC  TCGAGATTGAAGGACAGAAGAACAGG |
| P27 sh-F | CCGGTCGATTTTCAGAATCACAACTCGAGTTGT  GATTCTGAAAATCGATTTTTG |
| P27 sh-R | AATTCAAAAATCGATTTTCAGAATCACAACTCG  AGTTGTGATTCTGAAAATCGA |
| AMPK sh1-F | CCGGGTGACCTCACTTGACTCTTCTCTCGAG AG  AAGAGTCAAGTGAGGTCACTTTTTG |
| AMPK sh1-R | AATTCAAAAAGTGACCTCACTTGACTCTTCTCTC  GAGAGAAGAGTCAAGTGAGGTCAC |
| LKB1 sh1-F | CCGGGCAGCTGGTGGATGTGTTATCTCGAGATAA  CACATCCACCAGCTGTTTTTG |
| LKB1 sh1-R | AATTCAAAAACAGCTGGTGGATGTGTTATCTCGAG  ATAACACATCCACCAGCTG |
| LKB1 sh2-F | CCGGACAACGAAGAGAAGCAGAACTCGAGTTCTG  CTTCTCTTCGTTGTTTTTTG |
| LKB1 sh2-R | AATTCAAAAAACAACGAAGAGAAGCAGAACTCGA  GTTCTGCTTCTCTTCGTTGT |

**Raw images for original Western blots**

Figure 2g

KYSE150


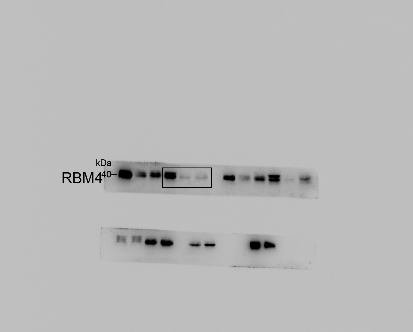






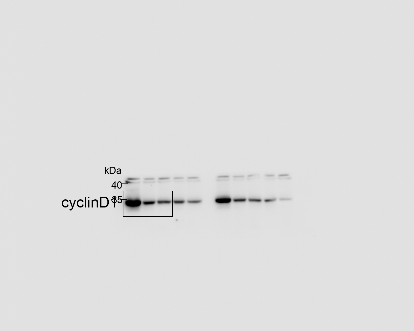

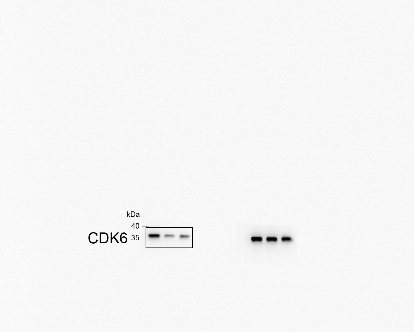

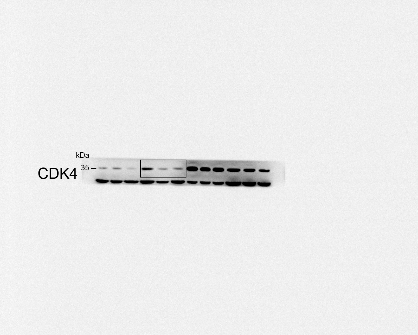


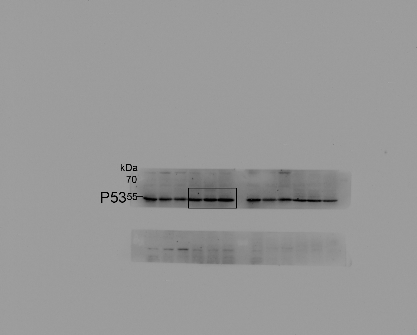

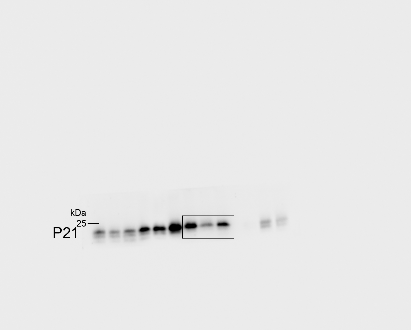

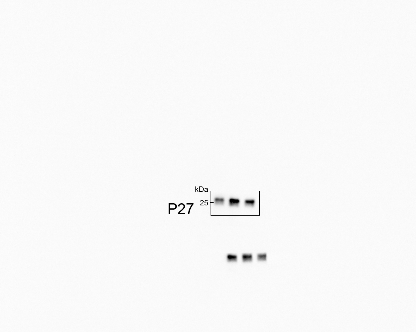


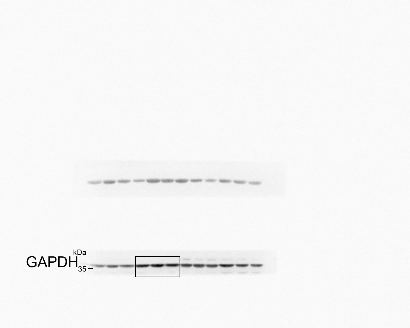


KYSE450


























Figure 3a

KYSE150













KYSE450













Figure 3e

KYSE150


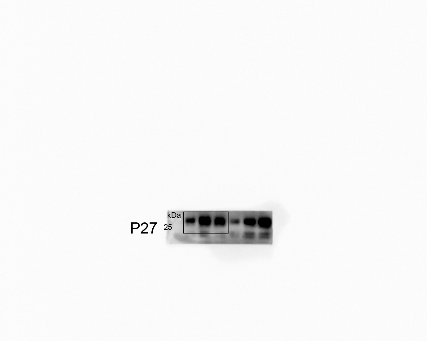

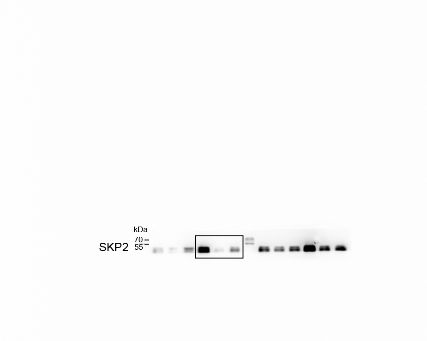

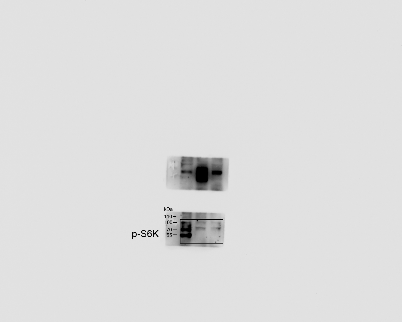


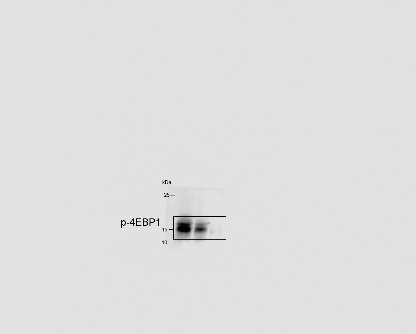

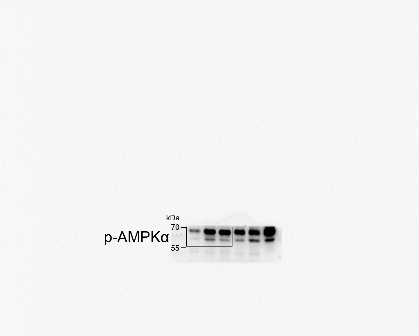

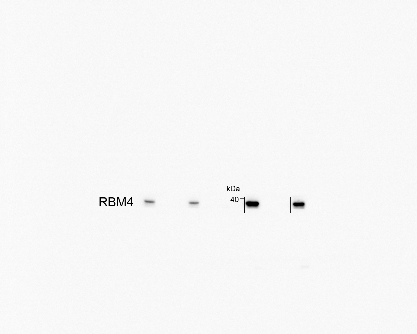


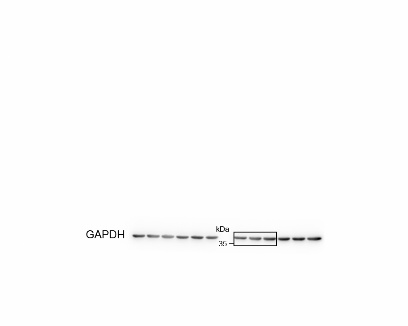


KYSE450


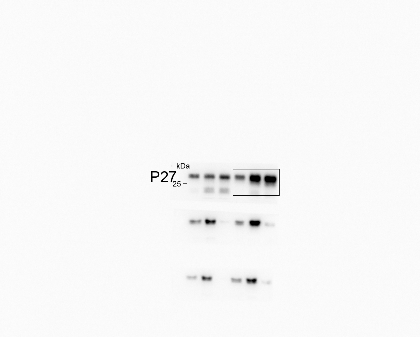

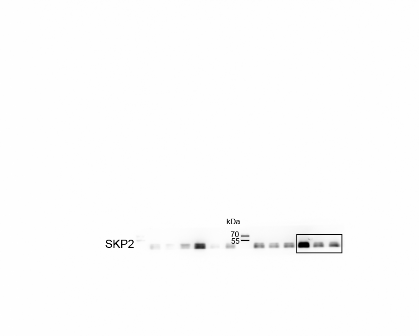

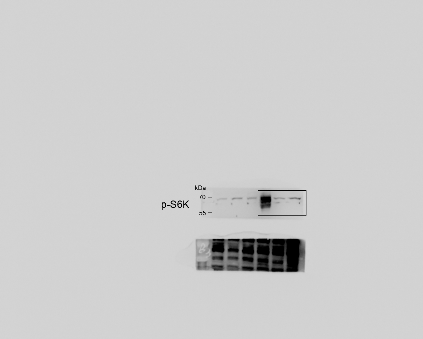


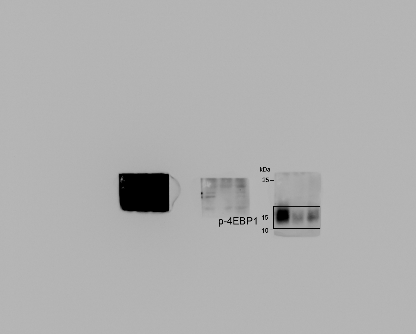

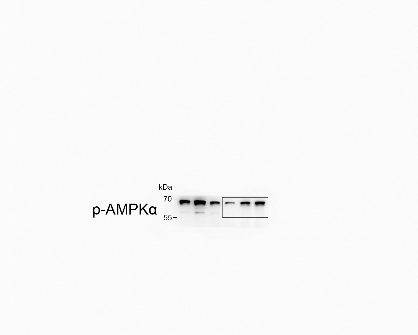

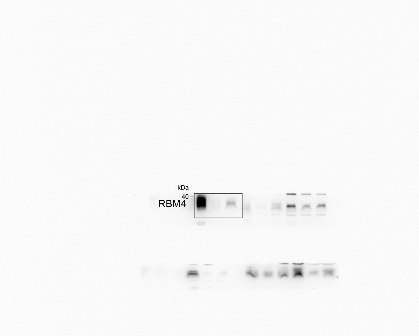


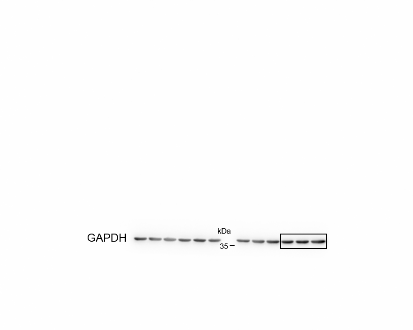


Figure 3f

KYSE150


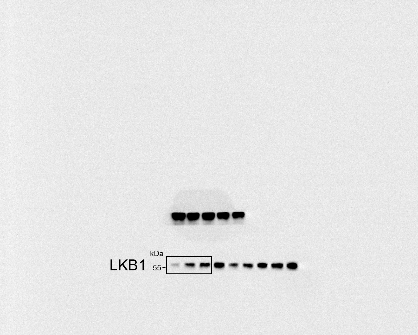

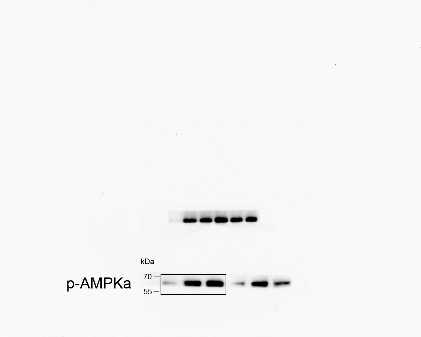

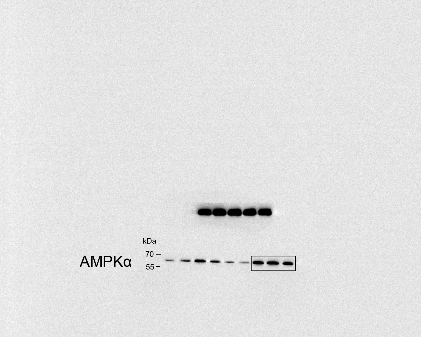

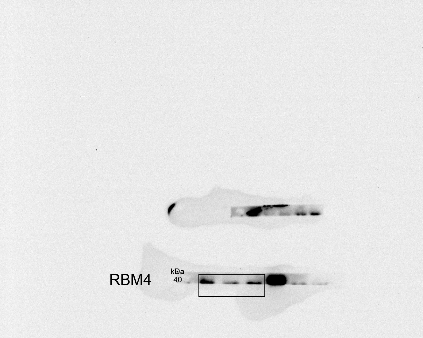

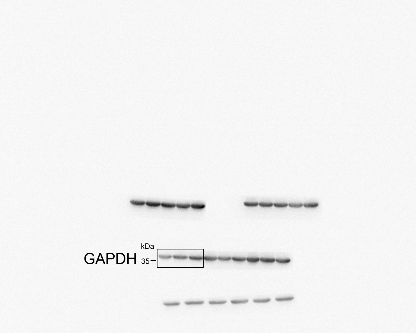


KYSE450


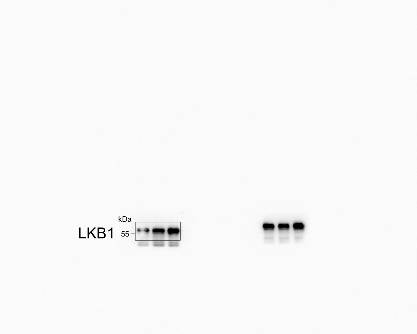

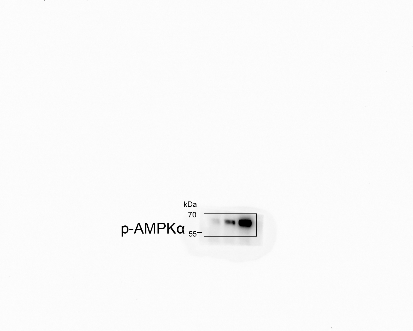

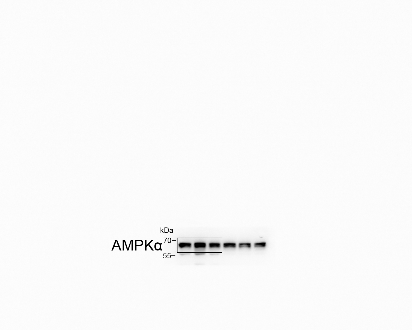

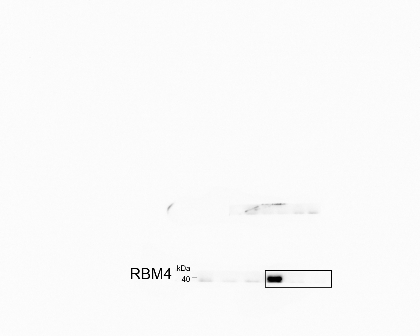

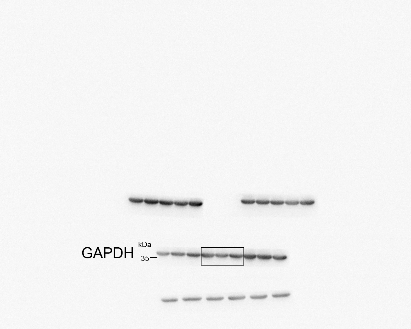


Figure 3g

KYSE150


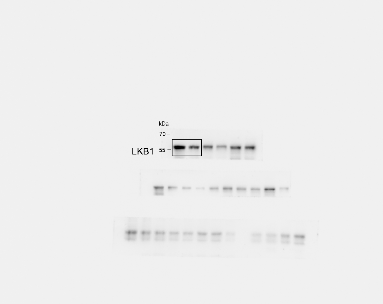

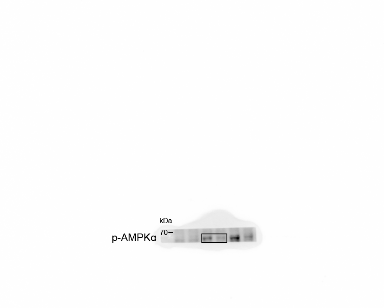

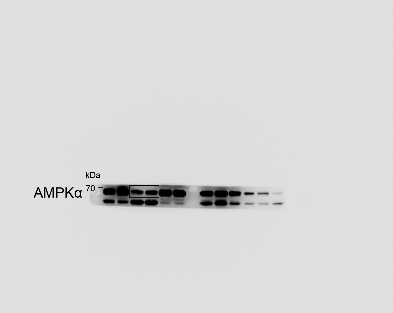

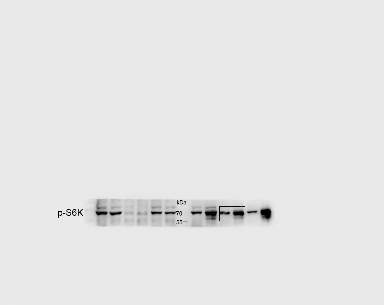



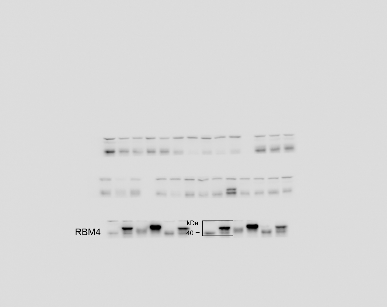

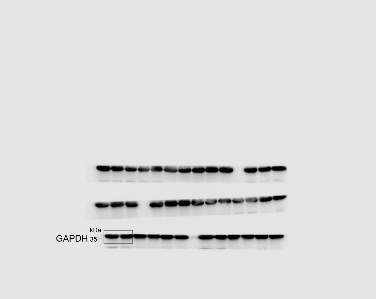


KYSE450


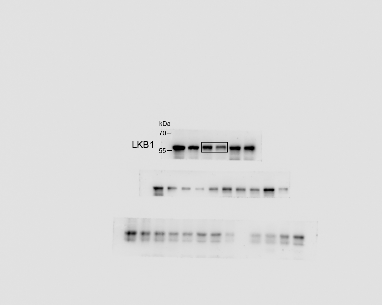

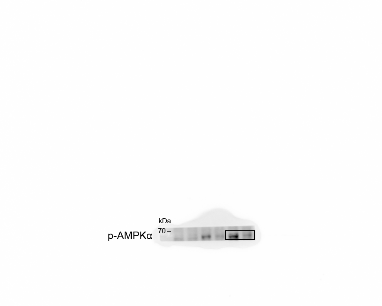

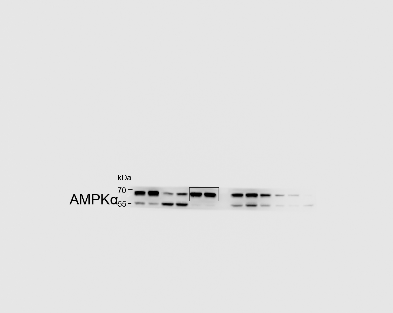

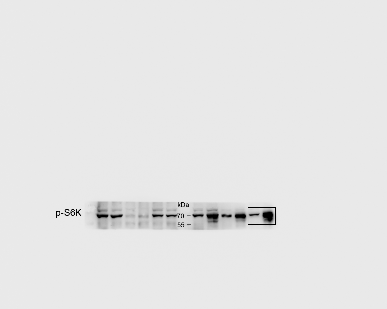

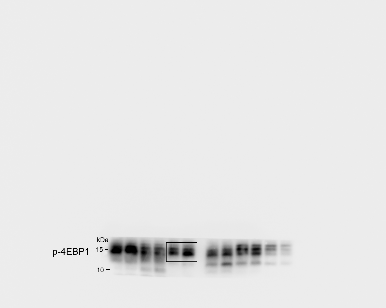

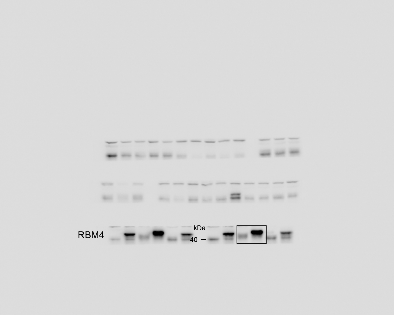

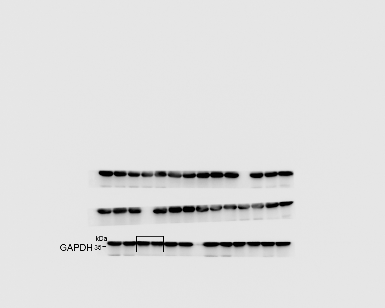


Figure 3h

KYSE150


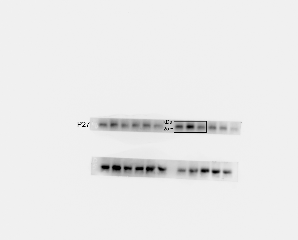

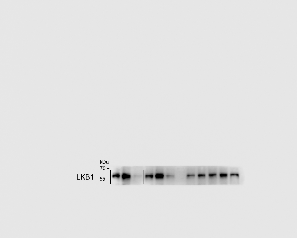

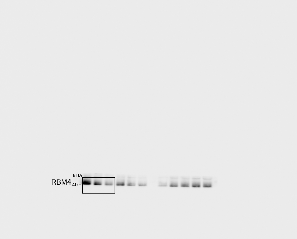

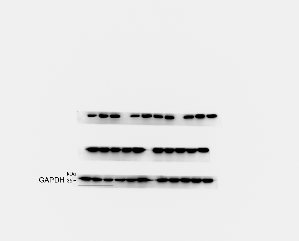


KYSE450


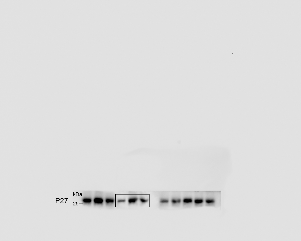

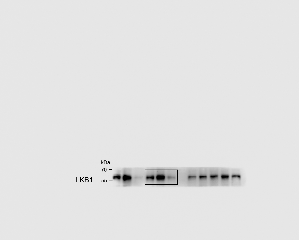

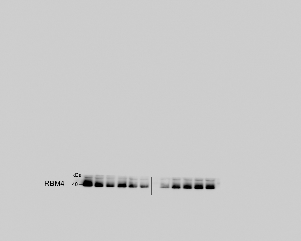

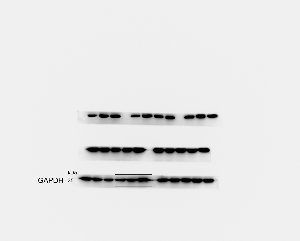


Figure 3i

KYSE150


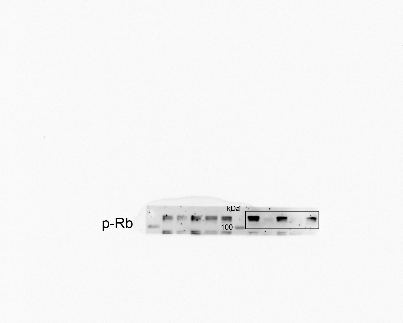

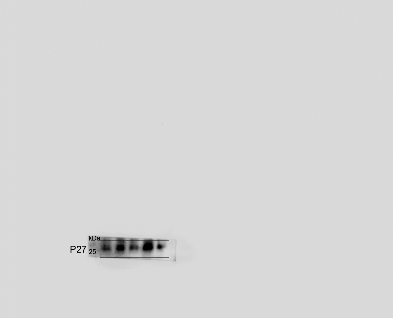

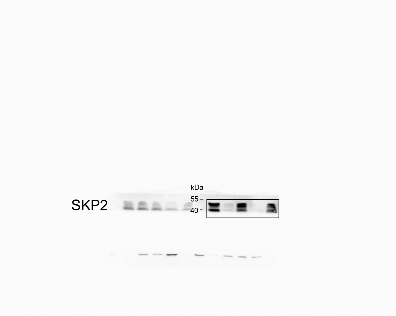


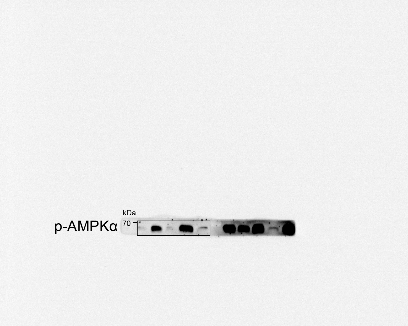

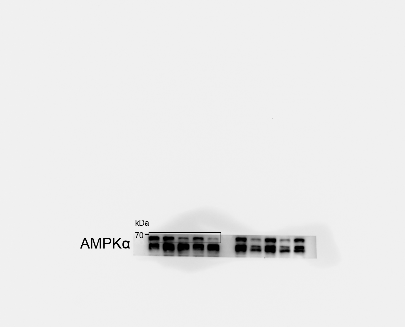

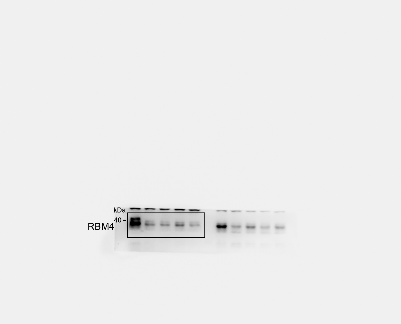


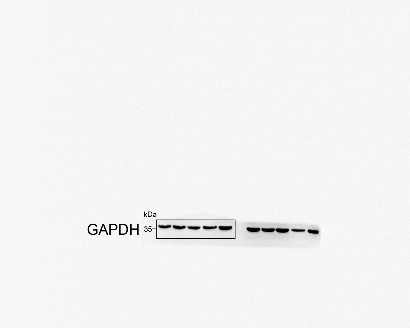


KYSE450


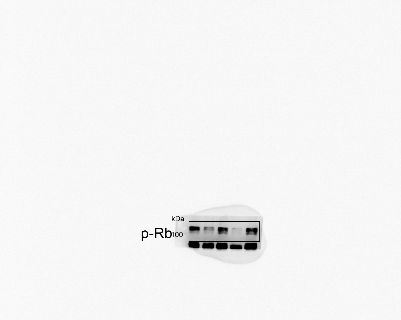

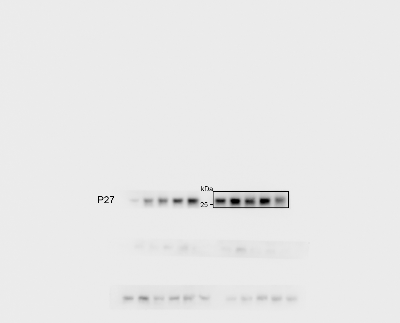

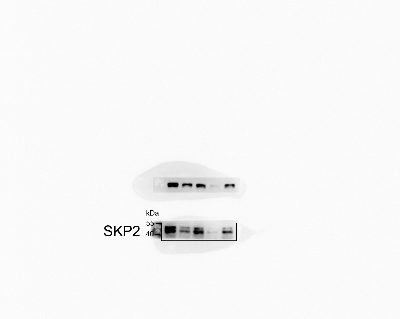


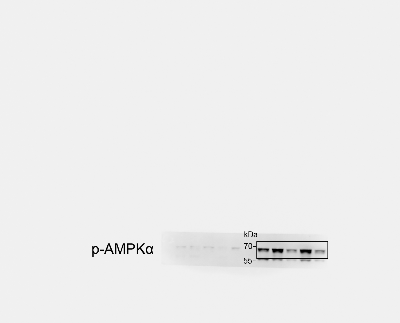

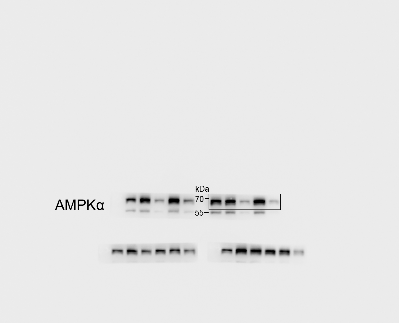

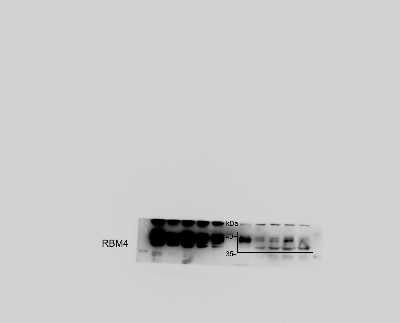


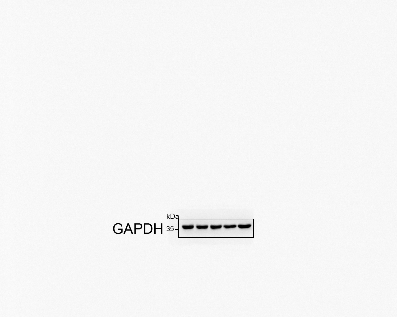


Figure 4a

KYSE150


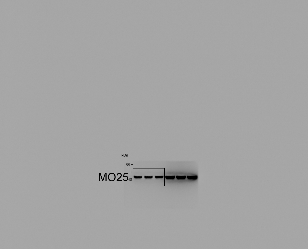

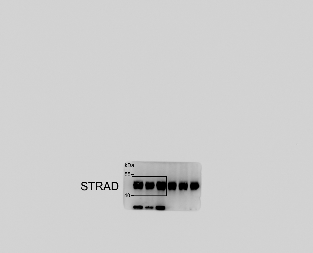

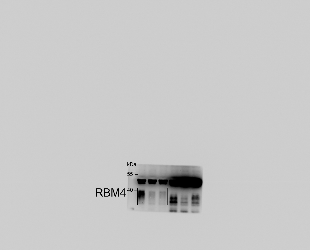

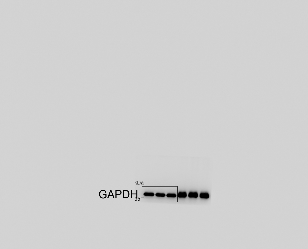


KYSE450

Figure 4b

KYSE150

KYSE450

Figure 4c(left)

Figure 4c(right)

Figure 4d

Figure 4e(left)

Figure 4e(right)

Figure 4f

Figure 4g

Figure 4h

Figure 5a

Figure 5b(left)

Figure 5b(right)

Figure 5c

Figure 5d

Figure 5e

Figure 5f

Figure 5g

Figure 5h

Figure 5j

Figure 7d(up)

KYSE70

KYSE30

KYSE150

KYSE450

KYSE510

Figure 7d(Down)

KYSE70

KYSE30

KYSE150

KYSE450

KYSE510

Figure 7e

Supplementary Figure 1c

KYSE150

KYSE30

KYSE450

Supplementary Figure 1d

KYSE150

KYSE30

KYSE450

Supplementary Figure 2a

KYSE30

KYSE150

Supplementary Figure 2b

KYSE30

KYSE150

Supplementary Figure 2e

KYSE30

KYSE150

NE2

Supplementary Figure 2f

KYSE30

KYSE510

Supplementary Figure 2h

KYSE150

KYSE450

Supplementary Figure 2j

Supplementary Figure 3a

KYSE30

KYSE150

Supplementary Figure 3b

KYSE30

KYSE150

Supplementary Figure 3e

Supplementary Figure 3f(up)

Supplementary Figure 3f(Down)

Supplementary Figure 3g

Supplementary Figure 4a

Supplementary Figure 4b

Supplementary Figure 4c(left)

Supplementary Figure 4c(right)

Supplementary Figure 4d(left)

Supplementary Figure 4d(right)

Supplementary Figure 4e

Supplementary Figure 4f

Supplementary Figure 4g

Supplementary Figure 6b

Supplementary Figure 6h(left)

Supplementary Figure 6h(right)

Supplementary Figure 6i

HT29(up)

HepG2(up)

HT29(Down)

HepG2(Down)
